# Cracking the code: how piRNA pathway shapes spermatogenesis and combats male infertility

**DOI:** 10.3389/fcell.2025.1657744

**Published:** 2025-09-22

**Authors:** Zhidan Hong, Sihan Huang, Li Li, Ying Gao, Binyu Ma, Qigang Fan, Yuanzhen Zhang, Mei Wang

**Affiliations:** ^1^ Center for Reproductive Medicine, Zhongnan Hospital of Wuhan University, Wuhan, Hubei, China; ^2^ Clinical Medicine Research Center of Prenatal Diagnosis and Birth Health in Hubei Province, Wuhan, Hubei, China; ^3^ Wuhan Clinical Research Center for Reproductive Science and Birth Health, Wuhan, Hubei, China; ^4^ Second Clinical Hospital of Wuhan University, Wuhan, Hubei, China

**Keywords:** PIWI proteins, spermatogenesis, male infertility, transposable elements, epigenetic regulation, piRNA biomarkers

## Abstract

Male infertility, accounting for approximately 50% of global infertility cases, is a growing concern in reproductive medicine. A fundamental cause lies in disrupted spermatogenesis—a complex, highly regulated process involving mitotic proliferation, meiotic division, and spermiogenic remodeling. Among the key regulatory pathways, PIWI-interacting RNAs (piRNAs) and their associated PIWI proteins have emerged as essential players in maintaining germline genome integrity and ensuring successful sperm development. However, their clinical relevance remain underexplored. This review provides a comprehensive synthesis of the piRNA pathway’s multifaceted roles across the full spectrum of spermatogenesis. We describe how piRNAs, together with PIWI proteins, silence transposable elements (TEs), guide chromatin remodeling, regulate mRNA translation, and protect sperm from environmental insults. We detail the stage-specific functions of piRNA machinery during spermatocytogenesis, spermatidogenesis, and spermiogenesis, supported by evidence from gene knockout models and cross-species studies. Particular emphasis is placed on piRNA biogenesis, including the primary processing pathway, the ping-pong amplification cycle, and terminal modifications mediated by enzymes such as PNLDC1 and TDRKH. Genetic disruptions in key piRNA pathway genes—including *MOV10L1*, *PNLDC1*, *SPOCD1*, and *TDRKH*—have been linked to clinical phenotypes such as non-obstructive azoospermia and severe oligozoospermia. We explore how these mutations impair piRNA maturation, compromise TE silencing, and trigger germ cell arrest, highlighting their diagnostic and therapeutic relevance. In addition, we discuss emerging applications of piRNAs as non-invasive biomarkers in seminal plasma, with altered piRNA profiles correlating with reduced sperm count and motility. Beyond pathogenesis, the piRNA pathway presents a promising frontier for reproductive interventions. We examine translational strategies targeting piRNA-associated proteins (e.g., RNF8-MIWI interaction modulators) and the potential for piRNA-guided gene silencing in germ cells. Moreover, we consider the impact of environmental toxins and epigenetic stressors on piRNA dynamics, suggesting new angles for fertility preservation. In summary, this review positions the piRNA pathway as a central regulator of male reproductive health. By integrating molecular biology with clinical genetics, we provide a roadmap for leveraging piRNA biology in the diagnosis, management, and treatment of male infertility.

## 1 Introduction

Infertility is a growing global health challenge, with the World Health Organization reporting a global infertility rate of 17.5%, with male factor accounting for 50% of cases (Cox et al., 2022). In China, the infertility rate has surged from 12% in 2007 to nearly 18% by 2020 (Qiao et al., 2021). Male infertility is fundamentally linked to spermatogenesis, a highly orchestrated event that produces functional sperm. Spermatogenesis is classically divided into three key stages: spermatocytogenesis, spermatidogenesis, and spermiogenesis. Disruptions in any of these stages—whether caused by genetic mutations, epigenetic alterations, or dysregulated non-coding RNAs—can impair sperm development and lead to infertility (Hosseini et al., 2024).

During spermatogonia formation, mammalian spermatogonial stem cells (SSCs) self-renew through niche signals in mice (Sasaki and Sangrithi, 2023). Subsequently, spermiogenesis involves chromatin condensation mediated by species-specific packaging proteins (protamines P1/P2 in humans, Transition nuclear protein1/2 (Tnp1/2) transition proteins in mice, and sperm nuclear basic proteins (SNBPs) in *Drosophila*) and morphological specialization, resulting in distinct sperm architectures such as hook-shaped murine heads and elongated *Drosophila* tails (Tirmarche et al., 2016; Qin et al., 2023; Subash and Kumar 2021). In mice, this process is classically divided into 16 steps based on nuclear and acrosomal morphology (Miyata et al., 2024). Steps 1-8 correspond to the round spermatid phase, characterized by Golgi-derived proacrosomal vesicle formation and flagellar assembly, while steps 9-16 involve elongation, nuclear condensation, and cytoplasmic remodeling to generate mature spermatozoa (Hess and Renato de Franca, 2008). The staging system differs across species; for example, human spermiogenesis is categorized into 6 phases rather than discrete steps, underscoring the necessity of species-specific annotations (Trost et al., 2023; Du et al., 2021). Finally, spermiation entails junction remodeling (e.g., a disintegrin and metalloproteinase 3 (ADAM3) in humans, lactate dehydrogenase A (LDHA) -dependent metabolic regulation in mice) to release mature sperm from Sertoli cells, with residual cytoplasm clearance varying across species (e.g., cytoplasmic droplets in humans *versus* (vs.) direct detachment in *Drosophila*) (Ribas-Maynou et al., 2021). Key interspecies divergences span cycle duration (∼74 days in humans vs. ∼10 days in *Drosophila*), metabolic pathways, and chromatin packaging strategies, as evidenced by recent studies on retinoic acid (RA)-driven SSC differentiation, fragile X-related protein 1 (FXR1) phase separation in translational activation, and protamine-linked infertility (Kang et al., 2022).

A major breakthrough in understanding spermatogenesis came with the discovery of PIWI-interacting RNAs (piRNAs) in 2006, identified in the germ cells of various organisms, including *Drosophila*, mice, and rats (Aravin et al., 2006; Aravin et al., 2001; Lau et al., 2006; Grivna et al., 2006; Girard et al., 2006; Vagin et al., 2006). piRNAs are a class of non-coding RNAs, ranging from 26 to 31 nucleotides (nt), slightly longer than miRNAs (22–24 nt) and siRNAs (20–25 nt). piRNAs interact with PIWI proteins—a specialized subfamily of the Argonaute (Ago) proteins named after *P-element Induced Wimpy testis* in *Drosophila*—forming the piRNA/PIWI complex (Li et al., 2025; Saito et al., 2006). This complex critically safeguards the genomic stability and fidelity of germ cells through targeted silencing of transposable elements (TEs), thereby preventing mutagenic disruptions caused by their aberrant activation (Vandewege et al., 2022; Kalmykova et al., 2005). It is also critical for gametogenesis, particularly in the differentiation and development of germ cells (Ramakrishna et al., 2021). Notably, piRNAs are predominantly derived from TE sequences located in specific genomic regions organized into arrays termed piRNA clusters (Gunawardane et al., 2007; Brennecke et al., 2007). These clusters are categorized as uni-strand or dual-strand clusters based on whether the transcription occurs from one or both DNA strands. Beyond TE silencing, piRNAs are involved in chromatin remodeling, RNA cleavage and stability, and the regulation of apoptosis, all of which are essential for proper germ cell development and spermatogenesis (Shoji et al., 2009; Carmell et al., 2007; Post et al., 2014; Aravin et al., 2006; Brennecke et al., 2007).

The piRNA pathway regulates both transcriptional and post-transcriptional events during spermatogenesis, ensuring the development of functional sperm (Wang et al., 2023b). Its main function is to ensure the suppression of TEs at both transcriptional and post-transcriptional levels, safeguarding genome stability. Transcriptional silencing mechanisms like DNA methylation and histone modifications (e.g., histone H3 lysine 9 di-methylation (H3K9me2)) ensure proper gene regulation and genome stability in spermatogenic cells, while post-transcriptional silencing mechanisms fine-tune mRNA stability in later stages (Legrand and Hobbs, 2018). In addition to protecting the genome, piRNAs are critical for the differentiation and maturation of germ cells (Claro-Linares and Rojas-Rios, 2025). Dysregulation of the piRNA/PIWI pathway has been implicated in various reproductive disorders, most notably male infertility. Studies have shown that mutations or dysfunctions within this pathway can lead to spermatogenic failure, further highlighting its importance in male reproductive health (Liu and Zhang, 2023). piRNA pathway dysregulation may also act as a key driver of oncogenic reprogramming in testicular germ cell tumors (TGCTs) through aberrant activation of proto-oncogenes, positioning this pathway as a potential multilevel biomarker for early detection and precision therapeutics (Wang D. et al., 2023).

This review provides an in-depth exploration of how piRNAs regulate spermatogenesis, focusing on their roles in TEs silencing, maintaining genomic integrity, and guiding germ cell differentiation. We investigate the mutations in piRNA pathway-related genes that lead to male infertility, shedding light on the underlying molecular mechanisms. Insights from knockout models will also be discussed to clarify the phenotypic and mechanistic impacts of piRNA-related gene deficiencies. Beyond understanding these molecular functions, we will explore the therapeutic potential of restoring piRNA function and leveraging gene editing techniques for male infertility treatment, while also highlighting the clinical application of piRNAs as diagnostic biomarkers. By integrating these findings, this review aims to advance the understanding of piRNA pathway and its potential in improving reproductive health outcomes.

## 2 piRNAs biogenesis and mechanism of action

### 2.1 piRNA biogenesis

In mammals, piRNAs are classified into two main types based on their expression during spermatogenesis: pre-pachytene piRNAs—first expressed in prospermatogonia, and pachytene piRNAs—expressed after the developing spermatocytes enter the pachytene phase of meiotic prophase I (Deng and Lin, 2002). Pre-pachytene piRNAs are primarily involved in *de novo* methylation and TEs silencing during embryonic development (Aravin et al., 2008). Pachytene piRNAs are predominantly derived from intergenic regions, 3′UTRs, pseudogenes, and repeat regions, which are thought to regulate mRNAs and lncRNAs via post-transcriptional gene silencing (Ortega et al., 2024).

Most piRNA sequences exhibit rapid evolutionary divergence across species, with minimal sequence conservation particularly between invertebrates and vertebrates (Shi et al., 2013; Chirn et al., 2015). However, mammals possess Eutherian-Conserved piRNA Cluster (ECpiC) loci whose expression demonstrates profound conservation across eutherian evolution. These conserved clusters maintain high expression in diverse mammals (e.g., humans, mice, dogs), suggesting specific adaptation to support eutherian reproductive functions (Chirn et al., 2015). The human piRNA pathway displays distinct evolutionary and functional features compared to other mammals. Despite syntenic conservation of pachytene piRNA loci in eutherians, human pachytene piRNA genes display striking evolutionary dynamics: promoter conservation contrasts sharply with accelerated sequence evolution in transcribed regions, resulting in population diversity indices surpassing most of the other genomic elements (Ozata et al., 2020). This accelerated evolution and hyper-diversity may facilitate differential target gene regulation, potentially influencing parental genome compatibility and thereby constituting a potential driver of reproductive isolation in eutherian lineages.

piRNA biogenesis occurs through two main pathways: the primary pathway and the ping-pong amplification cycle. The primary pathway can be separated into the transcription process and the phased piRNA process. In the transcription process, piRNA precursors are mostly originated primarily from specific genomic regions known as “piRNA clusters” or “piRNA-producing loci”, where they are transcribed by RNA polymerase II (Pol II) as long non-coding RNAs (Brennecke et al., 2007). Then in the phased piRNA process, RNA helicase Armitage activates the transport of Aubergine (Aub)-bound single-stranded piRNA precursors to the outer mitochondrial membrane. Later, piRNA precursors are cleaved by mitochondrial protein Zucchini/Phospholipase D6 (PLD6) into non-overlapping fragments, and polyadenylated at the 3′end. They are subsequently capped at the 5′ end and loaded onto PIWI proteins inside the nucleus, forming mature phased piRNAs with a uridine bias at the 5′ end (Gainetdinov et al., 2018; Brennecke et al., 2007; Mohn et al., 2015; Han et al., 2015). The ping-pong cycle represents a self-amplifying secondary biogenesis pathway majorly taking place in nuage. Cytoplasmic PIWI proteins (e.g., Aub and Ago3) recognize TEs via complementary base pairing and catalyze their endonucleolytic cleavage (Brennecke et al., 2007). The resulting cleavage fragments are subsequently captured by another cytoplasmic PIWI protein, processed into nascent piRNAs, thereby establishing a cyclical “cleavage-reloading” feedback loop (Gunawardane et al., 2007; Brennecke et al., 2007). This cycle plays a critical role particularly during the later stages of spermatogenesis in mice and in *Drosophila* germ cells (Wang et al., 2023c; Dai et al., 2019). The two pathways of piRNA biogenesis exhibit distinct functional: the primary pathway is responsible for *de novo* synthesis of the foundational piRNA pool, while the ping-pong cycle employs a self-amplification mechanism to efficiently suppress transposon activity. Their synergistic interaction ensures precise regulation of germ cell development and maintenance of genomic stability (Czech et al., 2018). The mechanism of primary pathway exhibits considerable evolutionary conservation, whereas secondary ping-pong amplification cycle displays marked interspecies divergence. Mammals and *Drosophila* employ the ping-pong amplification cycle for piRNA biogenesis, whereas *Caenorhabditis elegans* lacks this machinery. In *C. elegans*, piRNAs (termed 21U-RNAs) are directly transcribed by RNA polymerase II to form primary piRNAs. These primary piRNAs complex with target RNAs and recruit RNA-dependent RNA polymerases (RdRPs), catalyzing synthesis of secondary 22G-RNAs. These 22G-RNAs subsequently associate with worm-specific Argonaute (WAGO)-clade Argonaute proteins to execute post-transcriptional gene silencing (Pastore et al., 2022; Wang et al., 2023c). “piRNA trimming” is the final step of piRNA biogenesis, which enables the 3′ends of piRNA precursors to achieve their mature lengths. This process is mediated by enzymes such as poly(A)-specific RNase-like domain containing 1 protein (PNLDC1) and Tudor and KH domain-containing protein (TDRKH) (Saxe et al., 2013; Izumi et al., 2016). During piRNA biogenesis, cellular structures such as intermitochondrial cement (IMC), nuage and mitochondria-associated ER membranes (MAMs) play essential roles in TEs silencing and the stabilization of piRNA processing complexes (Aravin et al., 2009; Lim and Kai, 2007). The biogenesis pathway also ensures the protection of piRNAs from degradation through precise terminal modifications, including 3′-end trimming and 2′-O-methylation (Mohn et al., 2014; Zhang et al., 2014). These modifications, mediated by enzymes such as PolyA-specific ribonuclease PARN-1 (a piRNA trimmer) and Small RNA 2′-O-methyltransferase HENN-1 (a 2′-O-methyltransferase), are essential for piRNA stability and proper function. Deficiencies in these enzymes disrupt piRNA maturation, leading to 3′tailing, degradation, and compromised fertility (Pastore et al., 2021).

### 2.2 PIWI proteins and associated factors in the piRNA pathway

In humans, the PIWI protein family comprises four members: PIWIL1 (HIWI), PIWIL2 (HILI), PIWIL3, and PIWIL4 (HIWI2) (Li et al., 2024). In mice, the homologous proteins are designated as MIWI (PIWIL1), MILI (PIWIL2), and MIWI2 (PIWIL4) (Wei H. et al., 2024). The female-specific PIWIL3 is absent in mice and rats but can be identified in golden hamsters and bovine (Tan et al., 2020; Lv et al., 2023). Gene knockout mice of PIWI genes and associated factors in the piRNA pathway exhibit male fertility defects and varying degrees of spermatogenesis abnormalities, as shown in Table 1 (Dong et al., 2019; Bostick et al., 2007; Han et al., 2021; Morgan et al., 2019; Schopp et al., 2020; Yang et al., 2020; Vrettos et al., 2024; Lehtiniemi et al., 2022; Tan et al., 2021; Wei C. et al., 2024; Ding et al., 2017; Bolcun-Filas et al., 2011; Fu et al., 2016; Vourekas et al., 2015; Carmell et al., 2007; Deng and Lin, 2002; Kuramochi-Miyagawa et al., 2004; Fontaine et al., 2022; Yoshimura et al., 2018; Webster et al., 2005; Zoch et al., 2020; Wenda et al., 2017; Xiong et al., 2023). Notably, golden hamsters exhibit conserved expression patterns where PIWIL1 and PIWIL2 are predominantly cytoplasmic in prospermatogonia (Lv et al., 2023). PIWIL2 demonstrates dynamic co-localization with RNA-processing proteins mRNA-decapping enzyme 1A (DCP1A), Tudor domain-containing protein 1 (TDRD1), and mitochondrial ATP synthase F (1) complex subunit alpha (ATP5A) within both piP-bodies (a P granule that contains the PIWIL4-TDRD9 module) and IMC (Olotu et al., 2023; Wang X. et al., 2020). In contrast, PIWIL4 displays a dual subcellular distribution: cytoplasmic localization with DCP1A and Probable ATP-dependent RNA helicase DDX6 in piP-bodies coexists with nuclear accumulation, suggesting potential roles in both post-transcriptional regulation and chromatin-level processes (Lv et al., 2023).

**TABLE 1:** Phenotypes and Regulatory Mechanisms in piRNA-Related Gene Knockout Male Mice.

Genes	Knockout model	Fertility	Phenotypes	Regulatory mechanisms	Ref.
*Adad2*	KO mice	Infertile	Spermatogenesis is halted at the round spermatocyte stage with no prolonged spermatocytes	ADAD2 co-localizes with RNF17 in P-bodies, directing RNF17 to inhibit the ping-pong mechanism during pachytene piRNA biogenesis	Xiong et al. (2023)
*Ddx4*	KO mice	Infertile	*Ddx4* knockout male mice are infertile due to the complete loss of germ cells and abnormal testis development	DDX4 regulates piRNA loading onto MIWI2 by remodeling RNP complexes, ensuring transposon silencing and maintaining germ cell genomic stability	Wenda et al. (2017)
*Dnmt3l*	KO mice	Infertile	Male mice are viable but sterile, with germ cell loss and spermatocyte asynapsis, failing to reach pachytene	DNMT3L enhances DNMT3A/B activity, crucial for genomic imprinting, transposon silencing, and epigenetic regulation in germ cell development	Webster et al. (2005); Zoch et al. (2020)
*Gtsf1*	KO mice	Infertile	Male mice are sterile due to germ cell apoptosis after day 14, with meiocytes arrested before early meiosis	GTSF1 is a crucial factor for the slicing of target RNAs by PIWI-piRNAs and thus affects secondary piRNA biogenesis in prospermatogonia	Yoshimura et al. (2018)
*H3f3b*	KO mice	Infertile	Loss of *H3f3b* leads to progressive depletion of post-meiotic cells and finally cause infertility	Histone variant H3.3B regulates spermatogenesis by promoting piRNA transcription and facilitating X-chromosome inactivation	Fontaine et al. (2022)
*Mili*	KO mice	Infertile	Meiosis I is completely blocked at prophase, from zygotene stage to pachytene stage	Regulate DNA methylation, and silence the TE retrotransposons	Kuramochi-Miyagawa et al. (2004)
*Miwi*	KO mice	Infertile	The differentiation of round spermatids is arrested and the apoptosis is increased	Silence LINE1 retrotransponsons, regulate histone-protamine exchange and mRNAs stabilization	Deng and Lin (2002)
*Miwi2*	KO mice	Infertile	Meiosis I is completely blocked at prophase, from zygotene stage to pachytene stage, and spermatocytes exhibit an abnormal nuclear morphology	Regulate DNA methylation at TEs loci, and silence the TEs	Carmell et al. (2007)
*Mov10l1*	KO mice	Infertile	Mice are viable and healthy but show male sterility due to defects in spermatogenesis at early prophase of meiosis I	MOV10L1 processes piRNA precursors, loading them onto Piwi proteins to repress transposable elements and protect germline integrity	Fu et al. (2016); Vourekas et al. (2015)
*Mybl1*	KO mice	Infertile	Male mice are infertile, with reduced testis size and germ cell apoptosis due to meiotic arrest	MYBA regulates male meiosis by activating piRNA precursors and key piRNA metabolism genes like PIWIL1, ensuring proper piRNA biogenesis and function	Bolcun-Filas et al. (2011)
*Pnldc1*	KO mice	Infertile	Male mice are infertile, with reduced testis size and impaired spermiogenesis	PNLDC1 is a regulator of piRNA biogenesis, transposon silencing and spermatogenesis, protecting the germline genome in mice	Ding et al. (2017)
	cKO in spermatocytes	Infertile	Germ cells are primarily arrested at the elongated spermatid stage	PNLDC1’s exonuclease activity trims piRNAs, enabling postnatal piRNA-mediated LINE1 transposon silencing, critical for spermatogenesis and male fertility	Wei et al. (2024a)
*Rhox10*	KO mice	Infertile	*Rhox10* knockout mice exhibit male infertility due to a reduction in the number and function of SSCs	*Rhox10* regulates SSC self-renewal and differentiation by controlling genes essential for cell cycle and survival, ensuring spermatogenesis and male fertility	Tan et al. (2021)
	cKO in ProSGs	Infertile	*Rhox10*cKO in ProSGs causes infertility by impairing ProSG differentiation and migration, reducing SSCs	*Rhox10* upregulates piRNAs and *Piwil2* promoters, inducing PIWIL2 expression to inhibit LINE transposons, thereby maintaining genomic stability in germ cells	
*Rnf17* (*Tdrd4*)	KO mice	Infertile	Male mice are sterile, exhibit a complete arrest in round spermatids and fail to produce sperm	RNF17 regulates piRNA biogenesis by inhibiting the ping-pong cycle in P-bodies, ensuring their integrity and repressing transposable elements during spermatogenesis	Xiong et al. (2023)
*Smg6*	KO mice	——	Smg6 knockout mice exhibit embryonic lethality, with development arresting around embryonic day 7.5	SMG6 facilitates NMD, and its absence disrupts gene regulation essential for embryonic development	Lehtiniemi et al. (2022)
	cKO in early postnatal germ cells	Infertile	*Smg6*-cKO round spermatids fail to progress to form elongating spermatids	SMG6 regulates the male germline transcriptome by degrading NMD targets and coordinating with the piRNA pathway to control spermatogenesis and fertility	
*Spocd1*	KO mice	Infertile	Male mice are infertile with spermatozoa completely absent in the epididymis	SPOCD1 enhances MIWI2 and forms a *de novo* methylation complex with DNMT3L and DNMT3A for chromatin remodeling and transposon silencing	Zoch et al. (2020)
*Tdrkh*	KO mice	Infertile	Male mice are sterile due to defects in male meiosis	TDRD2 regulates transposon silencing by interacting with PIWI proteins in the piRNA pathway, thereby maintaining genomic stability in germ cells	Ding et al. (2017)
*Tdrd6*	KO mice	Infertile	Male mice are sterile, with a lack of elongated spermatids and distorted chromatoid body structure in round spermatids	TDRD6 mediates chromatoid body compaction through interactions with MIWI-NTRs, which are essential for the stabilization of spermiogenic transcripts	Vrettos et al. (2024)
*Tex15*	KO mice	Infertile	Male mice are infertile with reduced testis size and severe germ cell depletion due to meiotic arrest	TEX15 acts as an epigenetic regulator, functioning as a nuclear effector of MILI to silence transposable elements through DNA methylation	Schopp et al. (2020); Yang et al. (2020)
*Tut4*	KO mice	Fertile	*Tut4*-deficient animals are fertile, but combined *Tut4/7*-deficient animals show growth retardation and die perinatally	TUT4/7 mediates 3′mRNA uridylation, promoting the degradation of specific transcripts, thereby regulating cell cycle progression during male meiosis and ensuring proper spermatogenesis	Morgan et al. (2019)
	cKO in spermatocytes	Infertile	*Tut4/7*-cKO mice show atrophic testis and spermatogenesis stops at late pachytene stage		

**TABLE 1:** () Phenotypes and Regulatory Mechanisms in piRNA-Related Gene Knockout Male Mice.

Genes	Knockout model	Fertility	Phenotypes	Regulatory mechanisms	Ref.
	KO mice	Fertile	*Tut7*-deficient animals are fertile, but combined *Tut4/7*-deficient animals show growth retardation and die perinatally	TUT4/7 mediates 3′mRNA uridylation, promoting the degradation of specific transcripts, thereby regulating cell cycle progression during male meiosis and ensuring proper spermatogenesis	Morgan et al. (2019)
	cKO in spermatocytes	Infertile	*Tut4/7*-cKO mice show atrophic testis and spermatogenesis stops at late pachytene stage		
*Ubb*	KO mice	Infertile	*Ubb*-null male mice showed an azoospermia phenotype due to arrest of spermatogenesis at the pachytene stage	UBB ensures spermatogenesis by stabilizing piRNA-metabolic proteins and RNA-binding regulators via ubiquitin-dependent mechanisms	Han et al. (2021)
*Uhrf1*	KO mice	——	*Uhrf1* knockout mice exhibit embryonic lethality, typically around embryonic day 10.5	UHRF1 maintains DNA methylation, and its absence reduces methylation, causing genomic instability and abnormal embryonic development	Bostick et al. (2007)
	cKO in spermatogonia	Infertile	*Uhrf1* deficiency in mouse prospermatogonia causes spermatogonial stem cell loss, leading to Sertoli-cell-only syndrome and male infertility	UHRF1 could act as an alternative RNA splicing regulator and interact with Tle3 transcripts to regulate its splicing event in spermatogonia	Dong et al. (2019)
	cKO in spermatocyte	Infertile	Loss of *Uhrf1* in postnatal germ cells causes DNA hypomethylation, retrotransposon activation, DNA damage response, chromatin changes, and complete male sterility	UHRF1 suppresses retrotransposons and cooperates with PRMT5 and PIWI proteins in male germ cells	
	cKO in Sertoli cells	Infertile	Sertoli cell-specific *Uhrf1* knockout mice are completely sterile, with disrupted Sertoli cell proliferation, blood-testis barrier impairment, and immature germ cell sloughing	UHRF1 regulates the transcriptional program of ECM-related genes in Sertoli cells and establishes Sertoli cell-Germ cell crosstalk	

cKO, conditional knockout; KO, knockout; ProSGs, Prospermatogonia; SSCs, Spermatogonial Stem Cells; NMD, nonsense-mediated mRNA, decay.

During mammalian spermatogenesis, PIWI proteins exhibit stage-specific spatiotemporal dynamics that correlate with distinct regulatory functions. In early meiotic prophase I (leptotene/zygotene stages), PIWIL2 is exclusively expressed and localizes to nuage granules, ribonucleoprotein complexes critical for transposon silencing and mRNA surveillance (Gross, 2024). Upon transition to the pachytene stage, PIWIL1 emerges and forms dynamic co-localization clusters with PIWIL2 within these granules, suggesting synergistic roles in homologous recombination (Wang et al., 2022a).

Disruptions in PIWI proteins can lead to impaired piRNA biogenesis, negatively affecting spermatogenesis. *Piwil1*-deficient golden hamsters exhibit significantly reduced piRNA levels, causing spermatogenic arrest at the pachytene and zygotene stages, while *Piwil2* and *Piwil4* deficiencies result in almost complete loss of piRNA loss due to decreased mature gametes, leading to spermatogenic arrest during diplotene for most spermatocytes (Lv et al., 2023). Additionally, PIWI-Ins, a unique module within PIWI proteins, plays a crucial role in piRNA length selection. Deletion of PIWI-Ins in *Miwi* shifts MIWI(cytoplasmic) to load with shorter piRNAs, causing spermiogenic failure in mice (Wang et al., 2023b).

The proper functioning of PIWI proteins and associated factors, is essential for the precise completion of piRNA biogenesis. Mutations or defects in piRNA-related genes have also been shown to disrupt piRNA production and spermatogenesis, leading to male infertility. For a detailed discussion of these mutations in piRNA-related genes, please refer to Section 4.

## 3 piRNA in spermatogenesis: the regulatory network

**FIGURE 1:**
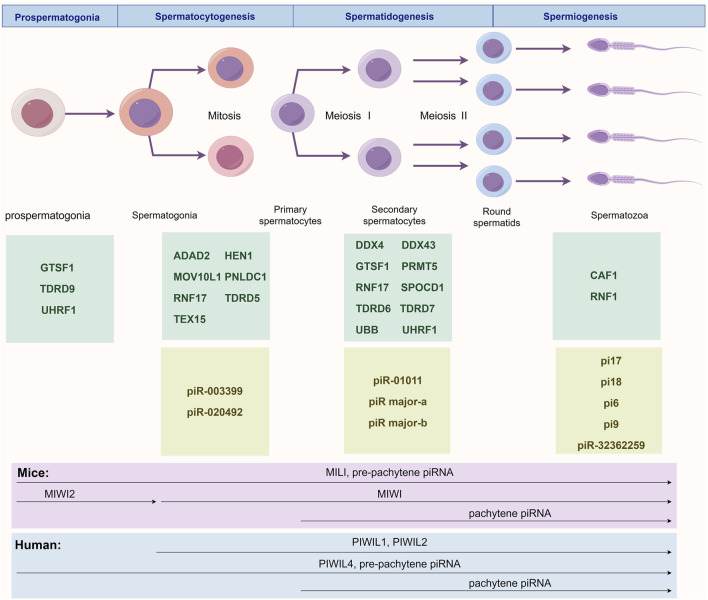
Stage-Specific Regulatory Network of the piRNA/PIWI Pathway During Spermatogenesis. This figure illustrates the major murine piRNAs and pathway proteins involved in prospermatogonia before birth and across three postnatal spermatogenic stages: prospermatogonia (GTSF1, TDRD9 and UHRF1), spermatocytogenesis (ADAD2, HEN1, MOV10L1, PNLDC1, RNF17, TDRD5, TEX15, piR-003399 and piR-020492), spermatidogenesis (DDX4, DDX43, GTSF1, PRMT5, RNF17, SPOCD1, TDRD6, TDRD7, UBB, UHRF1, piR-01011, piR major-a and piR major-b), and spermiogenesis (CAF1, RNF8, pi17, pi18, pi6, pi9 and piR-32362259). The lower panel depicts temporal expression patterns of PIWI proteins and piRNAs in humans and mice. In mice, MILI and pre-pachytene piRNAs appear in prospermatogonia and persist until spermiogenesis; MIWI2 is transiently expressed during prospermatogonia; MIWI expression begins postnatally and continues through spermatogenesis; pachytene piRNAs emerge at meiotic prophase I and remain until sperm maturation. In humans, PIWIL1 and PIWIL2 are expressed throughout postnatal spermatogenesis; PIWIL4 and pre-pachytene piRNAs are active from prospermatogonia through spermiogenesis; pachytene piRNAs follow the same temporal pattern as in mice.

**FIGURE 2:**
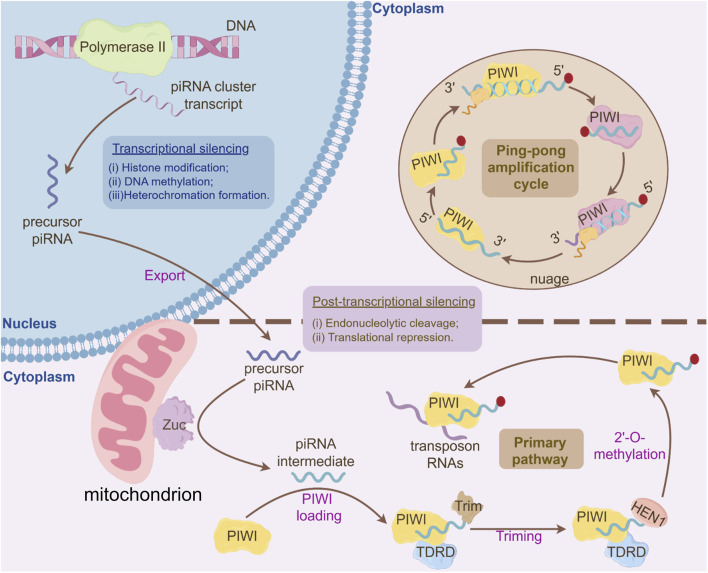
Integrated Mechanisms of piRNA Biogenesis and Gene Silencing Pathways. I. Primary piRNA Processing Pathway: Long single-stranded piRNA precursors, transcribed from piRNA clusters, are exported to the cytoplasm and processed into primary piRNA intermediates. These intermediates undergo 3′-to-5′ trimming by exonucleases to generate mature piRNA lengths. Subsequently, the 3′-ends of piRNAs are 2′-O-methylated by the HEN1 methyltransferase, stabilizing the piRNA molecules. The mature primary piRNAs are then loaded onto PIWI proteins, forming functional piRNA-PIWI complexes. These complexes recognize and bind complementary transposon RNAs in the cytoplasm, initiating downstream silencing mechanisms. II. Ping-Pong Amplification Cycle: The PIWI-piRNA complex binds and cleaves complementary transposon-derived RNAs in the cytoplasm, generating secondary piRNA fragments with a characteristic 10-nt overlap at their 5′ends. These secondary piRNAs are loaded onto PIWI, forming a self-amplifying loop that exponentially enriches piRNA populations targeting active transposons, ensuring robust silencing of transposable elements. III. Transcriptional Silencing in the Nucleus: Nuclear PIWI-piRNA complexes (e.g., PIWI in flies, MIWI2 in mammals) mediate epigenetic silencing through: (1) Histone Modification: Recruitment of histone methyltransferases depositing H3K9me3 marks, promoting heterochromatin assembly; (2) DNA Methylation: Guidance of DNA methyltransferases to target loci, establishing CpG methylation at transposon promoters; (3) Heterochromatin Formation: Cooperative action of H3K9me3-bound HP1 proteins and DNA methylation stabilizes condensed chromatin states, blocking transcriptional machinery access. IV. Post-Transcriptional Silencing in the Cytoplasm: Cytoplasmic PIWI/piRNA complexes bind transposon mRNAs, leading to: Endonucleolytic cleavage (“slicing”) of target RNAs, or Translational repression via recruitment of RNA degradation or inhibition factors.

The piRNA pathway is an intricate and highly specialized regulatory network that plays a crucial role in the proper development of male germ cells (Masone, 2024). Spermatogenesis, the process by which spermatogonia differentiate into mature spermatozoa, consists of three main stages: spermatocytogenesis, spermatidogenesis, and spermiogenesis. Throughout each stage, piRNAs, in conjunction with their associated PIWI proteins and piRNA pathway-associated proteins, perform vital functions that ensure genomic stability, regulate gene expression, and facilitate chromatin remodeling (Figures 1, 2).

This section explores how mutations in genes associated with the piRNA pathway contribute to male infertility in human, as summarized in Table 2 (Okutman et al., 2015; Akbari et al., 2019; Arafat et al., 2017; Zoch et al., 2024; Kherraf et al., 2022; Wyrwoll et al., 2022; Nagirnaja et al., 2021; Stallmeyer et al., 2024; Li et al., 2022). Additionally, findings from knockout mice models are examined to illustrate the phenotypic consequences and mechanistic insights of piRNA pathway-related gene deficiencies (Table 1). Therefore, we aim to offer a new perspective on elucidating the regulatory network of piRNA in spermatogenesis.

**TABLE 2:** piRNA-rRelated genetic mutations and their roles in human male infertility.

Mutant genes	Variants	Changes in amino acids	ACMG classification	Domain	Mutant phenotypes	Ref.
*PNLDC1*	c.1459C>T	p.Arg487Ter	VUS	outside the nuclease domain	non-obstructive azoospermia	Nagirnaja et al. (2021); Stallmeyer et al. (2024); Li et al. (2022)
	c.1387C>T	p.Arg463Ter	P	outside the nuclease domain	non-obstructive azoospermia; display different peak loss conditions in pachytene piRNAs	
	c.1174G>A	p.Glu392Lys	VUS	nuclease domain	non-obstructive azoospermia; significant decrease in piRNAs	
	c.809T>C	p.Leu270Pro	P	nuclease domain	non-obstructive azoospermia; display different peak loss conditions in pachytene piRNAs	
	c.776T>C	p.Tyr259His	P	nuclease domain	non-obstructive azoospermia; display different peak loss conditions in pachytene piRNAs	
	c.607-2A>T	abnormal splicing	P	near the nuclease domain	non-obstructive azoospermia; display different peak loss conditions in pachytene piRNAs	
	c.250C>T	p.Pro84Leu	VUS	upstream of the nuclease domain	non-obstructive azoospermia	
	c.172C>G	p.Leu58Val	P	nuclease domain	spermatogenic failure; severe oligoasthenoteratozoospermia	
	c.136dup	p.Ala46Glyfs	P	upstream of the nuclease domain	non-obstructive azoospermia	
*MOV10L1*	c.3268G>T	p.Glu1090Ter	VUS	outside the helicase domain	non-obstructive azoospermia; Sertoli cell-only phenotype	Stallmeyer et al. (2024); Li et al. (2022)
	c.3115G>A	p.Gly1039Ser	VUS	outside the helicase domain	non-obstructive azoospermia; Sertoli cell-only phenotype	
	c.3095_3098del	p.Pro1032fs	P	outside the helicase domain	non-obstructive azoospermia	
	c.2542G>A	p.Asp848Asn	VUS	outside the helicase domain	non-obstructive azoospermia	
	c.2447G>T	p.Gly816Cys	LP	DNA2/NAM7 helicase domain	non-obstructive azoospermia	
	c.2258T>C	p.Leu753Pro	VUS	DNA2/NAM7 helicase domain	azoospermia	

**TABLE 2:** () piRNA-rRelated genetic mutations and their roles in human male infertility.

Mutant genes	Variants	Changes in amino acids	ACMG classification	Domain	Mutant phenotypes	Ref.
	c.2179 + 3A>G	abnormal splicing	LP	outside the helicase domain	non-obstructive azoospermia; spermatogonial maturation arrest	
	c.743 + 5G>A	abnormal splicing	P	upstream of the helicase domain	azoospermia	
*PIWIL2*	c.1697G>A	p.Arg566Gln	VUS	PAZ domain	azoospermia	Stallmeyer et al. (2024)
	c.839A>C	p.Gln280Pro	VUS	PAZ domain	non-obstructive azoospermia; Sertoli cell-only phenotype	
*PIWIL1*	c.688C>T	p.Arg230Trp	LP	PAZ domain	non-obstructive azoospermia; round spermatid arrest	Stallmeyer et al. (2024)
*FKBP6*	c.832C>T	p.Arg278Cys	LP	TPR 3 repeat domain	extreme oligozoospermia	Stallmeyer et al. (2024); Wyrwoll et al. (2022)
	c.610C>T	p.Arg204Cys	LP	TPR 1 repeat domain	azoospermia; arrest of germ cells during meiosis	
	c.589-2A>G	abnormal splicing	P	near the TPR domain	extreme oligozoospermia; germ cell loss occurring in late meiosis and early spermiogenesis	
*HENMT1*	c.508_529dup	p.Phe177fs	P	TPR 1 repeat domain	extreme oligozoospermia	Kherraf et al. (2022)
	c.469-2A>T	abnormal splicing	LP	near the TPR domain	extreme oligozoospermia; no elongated spermatids	
	c.456C>G	p.Phe152Leu	P	Methyltransf_23	azoospermia	
	c.400A>T	p.Asn134Tyr	VUS	Methyltransf_23	non-obstructive azoospermia; round spermatid arrest	
	c.226G>A	p.Glu76Lys	P	Methyltransf_23	azoospermia	
*PLD6*	c.469del	p.His157fs	LP	PLD-like domain	non-obstructive azoospermia; Sertoli cell-only phenotype	Stallmeyer et al. (2024)
	c.1A>T	p.Met1Leu	VUS	upstream of the PLD-like domain	non-obstructive azoospermia; Sertoli cell-only phenotype	
*GPAT2*	c.1954C>T	p.Arg652Trp	LP	downstream of the GPAT domain	obstructive azoospermia; Sertoli cell-only phenotype	Stallmeyer et al. (2024)
	c.1879C>T	p.Arg627Ter	LP	downstream of the GPAT domain	obstructive azoospermia; Sertoli cell-only phenotype; spermatocyte maturation arrest	

**TABLE 2:** () piRNA-rRelated genetic mutations and their roles in human male infertility.

Mutant genes	Variants	Changes in amino acids	ACMG classification	Domain	Mutant phenotypes	Ref.
	c.1388C>T	p.Arg463Trp	VUS	downstream of the GPAT domain	obstructive azoospermia; Sertoli cell-only phenotype	
	c.1156-1G>A	abnormal splicing	LP	near GPAT domain	cryptozoospermia; spermatocyte maturation arrest	
	c.1130A>G	p.Thr377Ala	VUS	GPAT domain	obstructive azoospermia; Sertoli cell-only phenotype	
	c.146G>A	p.Ala49Thr	VUS	upstream of the GPAT domain	non-obstructive azoospermia; Sertoli cell-only phenotype	
*SPOCD1*	c.3354_3355insA	p.Gln1119fs	LP	Disordered region	non-obstructive azoospermia	Zoch et al. (2024)
	c.2912T>G	p.Leu971Arg	LP	SPOC domain	azoospermia	
	c.1991_1992del	p.Arg664fs	LP	TFIIS central domain	non-obstructive azoospermia	
*TDRKH*	c.1003A>T	p.Lys335Ter	P	Tudor domain	azoospermia	Kherraf et al. (2022)
*TDRD1*	c.887C>A	p.Ser296Tyr	LP	Tudor domain	non-obstructive azoospermia; spermatocyte maturation arrest	Stallmeyer et al. (2024)
*TDRD9*	c.3826G>T	p.Gly1276Cys	VUS	Tudor domain	cryptozoospermia	Stallmeyer et al. (2024); Arafat et al. (2017); Kherraf et al. (2022)
	c.3716 + 3A>G	abnormal splicing	LP	near Tudor domain	extreme oligozoospermia	
	c.3483_3484dup	p.Ser1162fs	P	Tudor domain	azoospermia	
	c.3148dup	p.Val1050fs	LP	Tudor domain	extreme oligozoospermia	
*TDRD12*	c.1243G>T	p.Ala415Ser	VUS	Helicase domain	extreme oligozoospermia	Stallmeyer et al. (2024)
	c.720_723del	p.Ser241fs	P	Helicase domain	non-obstructive azoospermia or cryptozoospermia; azoospermia	
	c.3157del	p.Leu1053fs	LP	Tudor domain	non-obstructive azoospermia; hypospermatogenesis	
	c.2432G>A	p.Arg811Gln	LP	Tudor domain	non-obstructive azoospermia; hypospermatogenesis	
	c.2419C>T	p.Arg807Trp	LP	Tudor domain	cryptozoospermia	
	c.986G>A	p.Arg329Gln	LP	Tudor domain	non-obstructive azoospermia; round spermatid arrest	

**TABLE 2:** () piRNA-rRelated genetic mutations and their roles in human male infertility.

Mutant genes	Variants	Changes in amino acids	ACMG classification	Domain	Mutant phenotypes	Ref.
	c.963 + 1G>T	abnormal splicing	LP	upstream of Tudor domain	non-obstructive azoospermia; spermatocyte maturation arrest	
	c.593A>G	p.Lys198Arg	VUS	upstream of Tudor domain	non-obstructive azoospermia; Sertoli cell-only phenotype	
	c.287A>C	p.Asn96His	VUS	N-terminal domain	non-obstructive azoospermia; Sertoli cell-only phenotype	
*ADCY10*	c.1205_1206del	p.His402fs	P	Catalytic domain	asthenozoospermia	Akbari et al. (2019)
*DDX4*	c.1532C>T	p.Pro511Leu	VUS	Helicase C-terminal domain	cryptozoospermia; round spermatid arrest	Stallmeyer et al. (2024)
*DDX25*	c.1129C>T	p.Arg377Trp	P	DEAD-box helicasedDomain	azoospermia	Kherraf et al. (2022)
*GTSF1*	c.221_222del	p.Arg74fs	P	Zinc finger domain	non-obstructive azoospermia; spermatocyte maturation arrest	Stallmeyer et al. (2024)
	c.97C>A	p.Ala33Glu	LP	near the N-terminal region	non-obstructive azoospermia; spermatocyte maturation arrest	
*MAEL*	c.908 + 1G>C	abnormal splicing	LP	near the MAEL domain	non-obstructive azoospermia; spermatocyte maturation arrest	Stallmeyer et al. (2024)
	c.799C>T	p.Arg267Trp	LP	MAEL domain	non-obstructive azoospermia; spermatocyte maturation arrest	
*TEX15*	c.3279T>G	p.Tyr1093Trp	P	ARM/HEAT repeat-like domain	spermatogenic failure associated with defects in meiosis	Okutman et al. (2015)

ACMG, stands for American College of Medical Genetics and Genomics. this organization provides guidelines for the classification of genetic variants, including categories such as P (Pathogenic), LP (Likely Pathogenic), and VUS (Variant of Uncertain Significance).

### 3.1 piRNA pathway in spermatocytogenesis

#### 3.1.1 piRNA pathway protects genomic stability during spermatocytogenesis

Spermatocytogenesis, the mitotic phase of spermatogenesis, involves the differentiation of diploid spermatogonia into primary spermatocytes. Genomic stability is crucial during this stage, as errors in DNA repair or chromosome segregation can lead to infertility or defective offspring (Xie et al., 2021). The piRNA pathway plays an indispensable role in maintaining genomic integrity in mice, primarily by silencing TEs and regulating gene expression.

TEs constitute a substantial portion of the genome and, if left unchecked, can lead to genomic instability. Overactivation of TEs results in DNA damage, apoptosis, sperm abnormalities, and infertility. Specifically, pre-pachytene piRNAs, which are activated during embryogenesis and remain active postnatally, direct the *de novo* methylation of repetitive elements, ensuring epigenetic silencing. In mice, nuclear PIWI proteins such as MIWI2 mediate transcriptional silencing of TEs, while cytoplasmic PIWI proteins, including MILI and MIWI, regulate post-transcriptional silencing via the ping-pong cycle (Manakov et al., 2015).

#### 3.1.2 PIWI proteins and their multifaceted roles in spermatocytogenesis

Deficiencies in PIWI proteins often lead to early arrest during spermatogenesis. In Atlantic salmon, Almeida et al. reported that the loss of *Piwil1* resulted in gametes deletion (Almeida et al., 2022). Some PIWI proteins also exhibit functional redundancy, in golden hamsters, PIWIL2 (cytoplasmic) can partially compensate for the absence of PIWIL4 in silencing specific TEs through the homotypic ping-pong pathway (Lv et al., 2023).

piRNA deficiencies also contribute to spermatogenic failure. In mice, Reddy et al. identified Pirmy and Pirmy-like RNAs as Y chromosome-derived noncoding transcripts that serve as templates for piRNAs, regulating genes critical for male fertility (Reddy et al., 2021). Notably, Y chromosome architecture and sequence content diverge markedly even among closely related species (e.g., *Mus musculus* vs. *Mus caroli*); thus, these findings may be context-specific to mice. Disruptions in these piRNAs, particularly due to Y chromosome deletions in the murine system, can cause abnormal sperm protein expression and male infertility (Reddy et al., 2021).

Deficiencies in the piRNA pathway exhibit species-specific phenotypic manifestations. Stallmeyer B et al. performed dual-species phenotypic analysis by characterizing individuals with biallelic high-impact variants in human piRNA pathway genes and generating corresponding knockout mouse models. Their investigation revealed fundamental interspecies divergence: defects in *PIWIL2*, *PLD6*, *Glycerol-3-phosphate acyltransferase 2 (GPAT2)*, and *TDRD12* produced profound phenotypic consequences in humans, whereas disruptions in *TDRD9*, *Protein maelstrom homolog (MAEL)*, and *PIWIL1* manifested more pronounced abnormalities in murine models. This differential phenotypic penetrance demonstrates that human and mice germlines possess distinct tolerance thresholds to impairments across the piRNA pathway machinery (Stallmeyer et al., 2024). “Trimming” is an important biochemical checkpoint in piRNA biogenesis, yet mutations in associated genes exhibit profound interspecies phenotypic divergence. Patients with *PNLDC1* mutations uniformly demonstrate spermatogenic arrest at the pachytene spermatocyte stage, with only rare round spermatid production—representing significantly earlier developmental arrest than the elongated spermatid-stage arrest observed in *Pnldc1* knockout mice. While murine models display severe molecular pathologies including profound reduction in MIWI-bound piRNAs and TE derepression, human *PNLDC1* mutants exhibit globally extended piRNA lengths without systematically documented TE dysregulation or downregulation in PIWI-bound piRNAs, despite arresting at an earlier spermatogenic stage (Stallmeyer et al., 2024). This species-specific molecular manifestation needs further investigation into the different compensatory pathways in piRNA biogenesis between human and mice.

The piRNA pathway silences TEs through IMC and piP-body complexes (Li et al., 2021; Aravin et al., 2009). Primary piRNAs bind to MILI in prospermatogonia, cleaving TE transcripts to generate secondary piRNAs, which are subsequently loaded onto MILI and MIWI2, enabling nuclear translocation for transcriptional silencing (Aravin et al., 2009). Interspecies divergence characterizes the piRNA pathway’s TE silencing mechanisms. Mice prospermatogonia primarily employ piRNA-guided post-transcriptional degradation for TE suppression, with DNA methylation playing a comparatively limited role (Inoue et al., 2017). Conversely, during meiosis, DNA methylation emerges as the dominant silencing mechanism under the guidance of piRNA pathway proteins including SPOC domain-containing protein 1 (SPOCD1) and MIWI2(nuclear) (Inoue et al., 2017; Kawase and Ichiyanagi, 2022; Zoch et al., 2020). In *Drosophila*, the piRNA pathway operates through fundamentally distinct regulatory architecture: the Rhino-Deadlock-Cutoff (RDC) complex orchestrates transcription of dual-strand piRNA clusters, enforcing TE silencing at the transcriptional level via histone modification-mediated heterochromatinization (Chen et al., 2021c; Zhang et al., 2021). In humans, piRNAs focus on regulating primate-specific retrotransposable elements, like SINE-VNTR-Alus (SVAs), exhibiting more complex targeting mechanisms than in mice (Fukuda et al., 2022). This phenomenon may be related to humans having a higher proportion of transposons compared to mice (Goodier, 2016; Banuelos-Sanchez et al., 2019).

#### 3.1.3 Key players MOV10L1, GTSF1, TEX15 and TDRD9 in piRNA-mediated regulation

MOV10L1, a testis-specific RNA helicase, serves as a master regulator of piRNA biogenesis. Loss of MOV10L1 function results in pre-pachytene piRNA deficiency, retrotransposon activation, and ultimately, spermatogenic failure and infertility (Fu et al., 2016; Vourekas et al., 2015). Non-obstructive azoospermia is characterized by testicular failure despite patent genital ducts (Agarwal et al., 2021). In non-obstructive azoospermia patients, mutations in *MOV10L1* have been linked to reduced piRNA levels and meiotic arrest (Wang et al., 2022b). MOV10L1 also interacts with ribosomes on primary piRNA transcripts, facilitating their translocation beyond stop codons, a process dependent on TDRD5 (Ding et al., 2018). A deficiency in MOV10L1 leads to primary transcripts accumulation and mature piRNA reduction, triggering retrotransposon activation and male infertility (Guan et al., 2021).

Additionally, MOV10L1 has a role in spermatogonia by silencing TEs through its RNA helicase activity and participates in miRNA-mediated regulation. In cytoplasmic RNA processing bodies (P bodies), MOV10L1 participates in post-transcriptional regulation (Zhu et al., 2015). A study by Fu et al. reported that MOV10 (MOV10L1’s paralog) deficiencies disrupted spermatogonial progenitor cells (SPCs), downregulating essential genes like *ETS translocation variant 5 (Etv5)*, *B-cell CLL/lymphoma 6 member B protein (Bcl6b)*, and *Zinc finger and BTB domain containing 16 (Zbtb16),* crucial for SPCs proliferation and self-renewal in mice germ cells (Fu et al., 2019). The disruption impairs SPCs’ ability to form seminiferous tubules, further emphasizing the importance of a fully functional piRNA pathway in spermatogenesis (Fu et al., 2019). Notably, in contrast to murine models, *Mov10l1* deficiency in golden hamsters results in infertility in both sexes, indicating a critical species-specific divergence in piRNA pathway functionality within female reproductive biology (Hasuwa et al., 2021; Loubalova et al., 2021).

Other proteins involved in the piRNA pathway also play crucial roles in genomic integrity. For instance, in mice, Gametocyte-specific factor 1 (GTSF1) and TDRD9 coordinate transcriptional silencing, with GTSF1 loss leading to depression of long interspersed element 1 (LINE-1)and intracisternal A particle (IAP) retrotransposons in prospermatogonia (Yoshimura et al., 2018). Testis-expressed protein 15 (TEX15), a testis-specific nuclear protein essential for the DNA damage response, works alongside MILI(nuclear) to recruit epigenetic silencing machinery to transposon loci (Yang et al., 2020). Notably, TEX15 is vital for spermatogenesis, but its deficiency does not disrupt piRNA biogenesis (Schopp et al., 2020). Finally, Adenosine deaminase domain-containing protein 2 (ADAD2), a protein highly expressed in male germ cells, regulates piRNA populations in mice. ADAD2 is a key player in the piRNA biogenesis interacting with multiple RNA-binding proteins, including MILI, MIWI, and RING finger protein 17 (RNF17). RNF17 preferentially binds to long precursors of pachytene piRNA clusters, and competitively reducing the opportunity for transposon RNA to interact with PIWI proteins, thereby preventing the over-activation of the ping-pong activity (Wasik et al., 2015). *Adad2* deficiency markedly reduces the formation of RNF17 granules, causing the aberrant activation of the piRNA ping-pong cycle, resulting in a higher proportion of secondary piRNAs with a 10th A bias. Deficiency in *Adad2* also results in elevated expression of piRNAs derived from transposons, while the cluster-derived pachytene piRNAs significantly decrease, including those associated with MILI and MIWI. This alteration closely resembles the abnormal piRNA patterns observed with RNF17 loss, suggesting a synergistic role of ADAD2 and RNF17 in regulating the genomic source preference of piRNAs (Lu et al., 2023).

Beyond maintaining genomic integrity, piRNAs also respond to testicular damage from environmental stressors. Chen et al. showed that heat stress in mice upregulates 88 piRNA clusters and downregulates 47 piRNA clusters (Chen et al., 2023). Key piRNA piR-020492 is significantly upregulated, affecting AMP-activated protein kinase (AMPK) and insulin pathways, inhibiting germ cell proliferation, and inducing apoptosis (Chen et al., 2023). Similarly, Zhang et al. found that exposure to Microcystin-LR (MC-LR) elevates piR_003399, causing cytotoxicity in spermatogonia in mice (Zhang et al., 2018). Suppressing piR_003399 increased Cyclin-dependent kinase 6 (CDK6) levels, improving sperm motility and cell cycle progression in mice (Zhang et al., 2018). piR_003399 levels in serum and plasma exhibited dose-correlated variations with MC-LR exposure, paralleling the severity of reproductive impairment in mice *in vivo* models (Zhang et al., 2018). This supports its utility as a potential circulating biomarker for MC-LR-induced reproductive toxicity. These findings highlight piRNAs’ critical role in protecting testicular health.

In conclusion, the piRNA pathway is indispensable for maintaining genomic stability during spermatocytogenesis by silencing TEs and regulating essential gene expression. Disruptions in this pathway can lead to severe spermatogenic failure and infertility.

### 3.2 piRNA pathway in spermatidogenesis

#### 3.2.1 piRNAs in maintaining genomic integrity and RNA cleavage

Spermatidogenesis, the second phase of spermatogenesis, involves the transformation of spermatocytes into spermatids through two meiotic divisions. This process begins with Meiosis I, where primary spermatocytes undergo chromosomal recombination and pairing during prophase, then divide into two secondary spermatocytes; Meiosis II follows, producing four haploid spermatids from each primary spermatocyte (Ishiguro, 2024). These spermatids are immature, round or oval-shaped cells lacking motility. The role of piRNAs in this phase is critical for maintaining genomic integrity and ensuring successful meiosis (Newkirk et al., 2017).

PIWI proteins play a central role in RNA cleavage, essential for reproductive function during meiosis. De Fazio et al. found that the RNA-cleaving activity of MILI relies on the conserved disulfide-directed β-hairpin fold (DDH) (Asp-Asp-His) motif in mice (De Fazio et al., 2011). Mutations in this motif (e.g., DAH or ADH) weakened MILI(cytoplasmic)’s cleavage activity, disrupting piRNA production, failing to silence TEs, and leading to spermatogenic arrest during meiosis. Co-factors, such as GTSF1, enhance MIWI(cytoplasmic)’s RNA cleavage function, while DEAD box polypeptide 4 (DDX4) and DDX43 promote the release of cleavage products, accelerating RNA degradation (Arif et al., 2022). Hsieh et al. further demonstrated that piRNAs, such as piR major-a and piR major-b, guide MIWI to cleavage sites, ensuring correct kinetochore assembly and chromosomal separation during meiosis in mice (Hsieh et al., 2020). Similarly, Chen et al. identified sex-specific piRNAs in *Drosophila melanogaster* that guide transcript cleavage, achieving gene regulation in the testis (Chen et al., 2021b).

Pachytene piRNAs play a central role in RNA cleavage. Cecchini K et al. demonstrated that the core function of murine pachytene piRNAs is to regulate target mRNAs through endonucleolytic cleavage, distinct from miRNA-like mechanisms or transcriptional silencing (Cecchini et al., 2024). Although the majority of these cleavage events do not alter the steady-state abundance of their target mRNAs, the regulation of a limited subset of critical mRNAs is indispensable for male fertility. Furthermore, transposon-derived piRNAs account for a higher proportion of targeting events, yet the high transcriptional activity of most targets buffers the impact of cleavage on their overall abundance. The evolutionary conservation of pachytene piRNAs is likely driven by selective advantages conferred by a minority of functional piRNAs, rather than the activity of the entire population—a feature potentially intrinsic to pachytene piRNA biology.

#### 3.2.2 TE silencing via piRNA-guided methylation and chromatin relaxation

The piRNA pathway enforces TE silencing via species-specific epigenetic mechanisms—including DNA methylation in mice and histone modifications in *Drosophila*—with disruptions causing germ cell arrest and male infertility. PIWI proteins collaborate with SPOCD1 to establish methylation-dependent silencing of retrotransposons (Zoch et al., 2020). In the murine system, SPOCD1 interacts with MIWI2 and recruits the DNA (cytosine-5)-methyltransferase 3-like protein (DNMT3L)-DNA (cytosine-5)-methyltransferase 3A protein (DNMT3A) methyltransferase complex to nascent transposon loci, facilitating repressive chromatin remodeling. While *de novo* DNA methylation is largely restricted to embryonic germ cell development, this machinery may primarily enforce maintenance methylation during spermatidogenesis in mice, ensuring persistent TE suppression. Zoch et al. demonstrated that SPOCD1 deficiency disrupts LINE1 and IAP silencing, leading to meiotic arrest and male infertility, despite intact piRNA biogenesis and MIWI2(nuclear) localization (Zoch et al., 2020).

Nuclear protein E3 ubiquitin-protein ligase UHRF1, a five-domain epigenetic regulatory factor, plays a pivotal role in coordinating the piRNA pathway with chromatin remodeling machinery (Dong et al., 2019). UHRF1 interacts with arginine methyltransferase Protein arginine N-methyltransferase 5 (PRMT5), which regulates histone arginine modifications (symmetric dimethylation of histone H4 on Arg 3 (H4R3me2s) and symmetric dimethylation of histone H3 at arginine 2 (H3R2me2s)) (Wang et al., 2015). UHRF1 also controls the localization of key PIWI pathway proteins (MILI, MIWI, and TDRKH), thereby regulating piRNA biogenesis in mice (Li et al., 2021). Dong et al. discovered that UHRF1 forms a complex with PRMT5 and MILI/MIWI in the cytoplasm of mouse spermatocytes, enhancing the cleaving activity of PIWI proteins, which contributes to the post-transcriptional suppression of retrotransposons (Dong et al., 2019). In *Uhrf1*-deficient mice, piRNA pathway dysfunction results in the loss of pachytene piRNAs, impaired cooperation with PRMT5, global DNA demethylation, retrotransposon upregulation, activation of the DNA damage response, and altered chromatin states, ultimately leading to male infertility (Dong et al., 2019).

The piRNA pathway exhibits interspecies divergence in its mechanisms for TE suppression. FKBP Prolyl Isomerase Family Member 6 (*Fkbp6*) has been implicated in secondary piRNA biogenesis and synaptonemal complex assembly in mice, with its deficiency leading to meiotic arrest (Xiol et al., 2012; Crackower et al., 2003). In murine models, *Fkbp6* loss disrupts synaptonemal complex formation and activates LINE-1 retrotransposons (Wyrwoll et al., 2022). In contrast, human cases of *FKBP6* deficiency also cause spermatogenic failure but lack the pronounced LINE-1 derepression observed in mice, suggesting that humans may employ more complex redundant mechanisms and fine-tuning capabilities for TE silencing within the piRNA pathway (Wyrwoll et al., 2022). This phenotypic divergence is similarly observed in *TDRD5* deficiency. While loss of *Tdrd5* in mice causes retrotransposon derepression and meiotic arrest (Yabuta et al., 2011), human testes harboring *TDRD5* mutations exhibit normal LINE-1 open reading frame 1 protein (LINE-1-ORF1p) expression with no observation of TE activation (Guo et al., 2025).

Notably, DNA methylation is not a universal TE silencing mechanism across species. For instance, in *Drosophila*, DNA methylation associated with the PIWI/piRNA pathway has not been reported. Instead, piRNA-mediated silencing relies predominantly on histone modifications (e.g., trimethylation of histone H3 lysine 9 (H3K9me3)) rather than DNA methylation (Zhang et al., 2021). Thus, the SPOCD1-MIWI2-DNMT3 axis described here reflects a murine-specific adaptation (Zoch et al., 2020). The uncharacterized protein C19ORF84 further bridges SPOCD1 with the methylation machinery, reinforcing piRNA-guided epigenetic silencing in mice. These findings underscore the importance of species context when interpreting piRNA-mediated TE control mechanisms (Zoch et al., 2024).

TE silencing mediated by piRNAs may also be achieved via chromatin modification pathways during spermatidogenesis. Research by Mahadevan et al. highlighted the role of histone H1t in chromatin regions containing TEs, where it interacts with repressive epigenetic markers and proteins, suggesting a mechanism for piRNA-mediated recruitment of repressive chromatin factors in mouse pachytene spermatocytes (Mahadevan et al., 2020). Under the regulation of piRNAs, H1t might facilitate the suppression of TEs through the induction of localized chromatin relaxation, which enables the recruitment of heterochromatin-associated proteins and repeat repressive-associated protein factors, ultimately leading to the formation of closed chromatin repressive structures (Mahadevan et al., 2020). Additionally, piRNA clusters are enriched with the histone variant H3.3B, which is crucial for spermatogenesis. Loss of *H3f3b* in mice severely impairs piRNA cluster transcription during meiosis, leading to infertility due to defective post-meiotic cells (Fontaine et al., 2022).

#### 3.2.3 piRNAs-mediated mRNA translation and apoptosis regulation

During spermatidogenesis, piRNAs also guide mRNA translation. Dai et al. identified specific MIWI-CLIP clusters on piRNA target sites in key genes like *Plectin*, *Arf-GAP domain and FG repeat-containing protein 1 (Agfg1)*, *TATA box-binding protein-like 1 (Tbpl1)*, *CCR4-NOT transcription complex subunit 4 (Cnot4)*, and *Ubiquitin-like protein ATG12 (Atg12)*, which are crucial for acrosome formation and spermatid development in mice (Dai et al., 2019). These mRNAs contain AU-rich elements (AREs) in their 3′UTRs, necessary for piRNA-mediated translational activation. This process also inhibits the repressive actions of specific piRNAs, like piR_010111, on some genes. piRNA-mRNA interactions guide MIWI binding to these loci, assembling the MIWI/eIF3f/HuR complex to promote translation. Wang et al. further demonstrated that extended piRNA-mRNA complementarity in mice enhances RNA-binding protein human antigen R (HuR) binding to target mRNA in an ARE-independent manner, expanding the regulatory scope of the piRNA pathway (Wang et al., 2023b).

The interaction between piRNAs and the ubiquitin-proteasome system (UPS) represents an emerging area of research, particularly in the contexts of germline development and cancer. The piRNA pathway has been linked to post-translational protein degradation and apoptosis—the ubiquitin (Ub) pathway. Ub is a highly conserved protein crucial for fertility in both male and female mice (Martin-Villanueva et al., 2021). In mice, the polyubiquitin gene *Polyubiquitin-B* (*Ubb)* is composed of a tandem repeat of four ubiquitin-coding units; its primary function is to maintain cellular ubiquitin homeostasis by encoding tandemly repeated ubiquitin units and regulate critical physiological processes (Martin-Villanueva et al., 2021). Further mechanistic studies demonstrated that *Ubb* deficiency disrupts ubiquitin homeostasis, triggering a cascade of pathological events: (i) depletion of the free ubiquitin pool impairs proteasomal degradation, resulting in abnormal protein accumulation; (ii) dysregulation of germline-specific genes (Ddx4, Tdrd6, Tdrd7, Rnf17) and piRNA pathway effectors (MIWI, MILI) compromises post-transcriptional regulation; and (iii) synergistic dysfunctions culminate in pachytene-stage meiotic arrest, germ cell apoptosis, and complete gametogenesis failure (Han et al., 2021). Charmant O et al. revealed that the ubiquitination of the *Paramecium* PIWI protein is indispensable in male fertility. Ptiwi09 is predominantly regulated by Gtsf1 (Charmant et al., 2025). This process is initiated when Gtsf1 interacts with the Ptiwi09-maternal polyploid somatic macronucleus (MAC)-scnRNA complex, which is paired with nascent non-coding transcripts from the MAC. This binding triggers Ptiwi09 ubiquitination, facilitating the degradation of both the Ptiwi09 protein and its associated MAC-scnRNAs, compromising genome integrity. In Gtsf1-KD *Paramecium*, a marked reduction in Ptiwi09 ubiquitination is observed, resulting in abnormal MAC-scnRNA levels and TE activation (Charmant et al., 2025).

Small ubiquitin-like modifier (SUMO) is a highly conserved post-translational modifier that expands the functional diversity of the eukaryotic proteome through dynamic conjugation to target proteins (Celen and Sahin, 2020). Recent studies have revealed an intimate mechanistic link between SUMOylation and the piRNA pathway in female reproductive system. In *D. melanogaster*, diGly-based proteomic profiling has uncovered widespread SUMOylation of core piRNA pathway components, including nuclear factors such as Piwi and Panoramix (Panx), as well as cytoplasmic nuage constituents like Spindle-E (Spn-E) and Mael (Ninova et al., 2023). Notably, Piwi differentially regulates the SUMOylation status of these substrates, suggesting a hierarchical SUMO-dependent architecture within the piRNA pathway (Ninova et al., 2023). Functional ablation of SUMO disrupts the integrity of nuage, transforming perinuclear Vasa- and Aub-positive granules into dispersed puncta, thereby impairing piRNA biogenesis and post-transcriptional silencing of TEs (Ninova et al., 2023). Parallel work done in hermaphrodite *C. elegans* has demonstrated that SUMOylation of the germline determinant pharynx and intestine in excess protein 1 (PIE-1) is also essential for both germline fate maintenance and piRNA-mediated TE repression, underscoring the evolutionary conservation of this regulatory axis (Kim et al., 2021).

Mechanistically, SUMOylation acts as a molecular switch linking piRNA-guided silencing to chromatin remodeling. Ninova M et al. discovered that in *Drosophila*, the SUMO E3 ligase Su(var)2-10 interacts directly with the Piwi–Panx–Arx complex and facilitates the SUMOylation of both itself and associated chromatin factors (Ninova et al., 2020). This modification promotes recruitment of the SetDB1/Windei histone methyltransferase complex, enabling deposition of H3K9me3 marks at transposon loci and transcriptional repression of approximately 60% of TE families (Ninova et al., 2020). These findings establish SUMOylation as an essential scaffold for chromatin-based transposon control, effectively coupling small RNA recognition to epigenetic enforcement.

While the role of SUMOylation in piRNA-mediated silencing is well established in model organisms, its relevance in mammals and its interplay with other post-translational modifications are less clear. Although the role of SUMO in the piRNA pathway has been well studied in the female reproductive system, its function in male germ cells requires further investigation. Additionally, the dynamic regulation of SUMOylation and deSUMOylation, and their impact on piRNA pathway and condensate formation, are in need of further exploration.Taken together, the piRNA pathway is crucial for spermatidogenesis, maintaining genomic integrity, controlling protein degradation, regulating mRNA translation and apoptosis—all essential for the proper development and maturation of spermatids.

### 3.3 piRNA pathway in spermiogenesis

#### 3.3.1 piRNA’s role in TE silencing during spermiogenesis

Spermiogenesis, the final stage of spermatogenesis, exhibits marked species-specificity in its morphological and molecular progression. This stage contains key molecular events including: proacrosomal vesicle formation, histone-to-protamine transition, acrosome biogenesis flagellar assembly, spermiation and epididymal maturation (Hess and Renato de Franca, 2008). The piRNA pathway and its functional partners regulate critical aspects of murine spermiogenesis, including transposon silencing during nuclear condensation and mRNA surveillance in cytoplasmic droplets.

The primary biological function of piRNAs in spermiogenesis is TEs silencing, particularly LINE1 (L1) retrotransposons, which make up about 20% of the mammalian genome (Doucet et al., 2015). L1 retrotransposition involves RNA polymerase II (RNAPII) synthesizing L1 RNA, which is then cleaved and polyadenylated. The transcript is exported to the cytoplasm and translated into two proteins: ORF1 and ORF2. ORF1 packages L1 RNA for nuclear import, while ORF2 facilitates reverse transcription and integration of L1 into the genome (Miyoshi et al., 2019). piRNA-mediated silencing of L1 is crucial for preventing spermatogenic arrest and infertility in male germ cells (Yang and Wang, 2016). In *Pnldc1*-deficient mice, piRNA trimming is disrupted, leading to L1 depression and spermatogenic arrest (Ding et al., 2017). TDRKH and PNLDC1 modulate MIWI localization in chromatoid bodies (CBs), promoting L1 silencing in round spermatids (Wei C. et al., 2024). Mutations in the *Rhox* gene cluster, encoding transcription factors critical for reproductive health, have also been linked to L1 silencing (Tan et al., 2021) (Table 1).

In *C. elegans*, the cytoplamsic Argonaute protein CSR-1 antagonizes piRNA-mediated silencing by promoting the expression of neo-formed RNAs, protecting germline-expressed genes (Cornes et al., 2022). This regulation facilitates the precise timing of piRNA silencing functions during spermiogenesis. piRNA biogenesis and degradation are tightly controlled, as seen in silkworms, where *small RNA 2′-O-methyltransferase (BmHen1)* and *BmPnldc1* are essential for piRNA stability and TE silencing (Yang et al., 2022).

#### 3.3.2 piRNA-mediated chromatin remodeling, histone-protamine transition and cytoplasmic exclusion

Chromatin remodeling, particularly the histone-to-protamine transition, is another critical process regulated by the piRNA pathway. PIWI proteins, in cooperation with piRNAs, regulate histone modifications during spermiogenesis. In *Drosophila*, CDS-piRNAs regulate genes involved in histone acetylation, such as *newly excysted juveniles (nej)*, affecting histone acetyltransferase activity (Iki et al., 2023). Loss of *aubergine(aub)*, a PIWI-interacting protein, leads to excessive histone acetylation and disrupted histone-protamine transitions, resulting in defective spermiogenesis (Iki et al., 2023). The THO RNA export complex and *Gtsf1* are also involved in piRNA-guided chromatin modifications, such as H3K9me3, to ensure TE silencing during spermatogenesis in *Drosophila* (Donertas et al., 2013).

The elimination of PIWI proteins is closely linked to histone ubiquitination. Specifically, MIWI and RNF8 regulate H2B ubiquitination and MIWI degradation, both of which are essential for completing spermatogenesis in mice (Gou et al., 2017). MIWI acts as a molecular switch, initiating the histone-to-protamine transition critical for sperm maturation. Disruption of MIWI or RNF8 expression leads to accumulation of MIWI in cytoplasm and male infertility due to abnormal sperm morphology. piRNAs mediate PIWI clearance to ensure successful spermatogenesis (Gou et al., 2017). Zhao et al. found that in late-stage mouse spermatids, MIWI is degraded via the anaphase promoting complex/cyclosome (APC/C)-26S proteasome pathway, and piRNAs facilitate the interaction of MIWI with APC/C substrate subunits. This piRNA-triggered de-ubiquitination and MIWI degradation promote piRNA clearance, aiding sperm maturation (Zhao et al., 2013).

Nuclear condensation and cytoplasmic exclusion represent critical terminal events in spermiogenesis, essential for functional sperm maturation. Nuclear compaction achieves dramatic volumetric reduction of the spermatid nucleus through hyper-condensation of chromatin, forming the streamlined sperm head architecture. Concurrently, cytoplasmic exclusion eliminates superfluous organelles and transcripts via sequestration into residual bodies, purging most of cytoplasmic volume (Hess and Renato de Franca, 2008). This concerted remodeling enhances sperm motility while eliminating redundant mRNAs/proteins that could compromise early embryogenesis. Pachytene piRNAs provide the molecular logic underpinning these transformations. Through pi-RISC mediated large-scale mRNA clearance, they terminate dispensable cellular programs, enabling structural reorganization. Gou LT et al. demonstrated that murine pachytene piRNAs form chromatin assembly factor 1 (CAF1)-containing pi-RISCs in elongating spermatids. These complexes employ imperfect base-pairing to bind 3′UTRs of target mRNAs (e.g., G protein-coupled receptor kinase 4 (Grk4), Transcription factor SOX-6 (Sox6)), where CAF1 catalyzes deadenylation-independent decay. Crucially, this degradation operates independently of MIWI’s slicer activity, but requires MIWI’s piRNA-loading competence (Gou et al., 2014). By orchestrating transcriptome-wide silencing, pachytene piRNAs facilitate nuclear compaction and cytoplasmic exclusion–establishing the molecular framework for spermatid-to-spermatozoon transformation.

#### 3.3.3 piRNA pathway’s impact on sperm motility and morphology

The piRNA pathway is also responsive to environmental factors that affect chromatin remodeling. Liu et al. demonstrated that exposure to silicon dioxide nanoparticles (SiNPs) alters MIWI expression patterns in mice spermatocytes, particularly in round spermatids (Liu et al., 2021). This increased MIWI expression can cause RNF8 retention in the sperm cell cytoplasm, leading to downregulated histone (H2A/H2B) removal and inhibited formation of ubH2A and ubH2B (Liu et al., 2021). The disrupted exchange of histone-to-protamine and chromatin condensation ultimately affecting the round spermatid differentiation and leading to sperm abnormalities (Liu et al., 2021). The study offers insights into how environmental contaminants like SiNPs negatively impact reproductive health. The piRNA pathway is essential for acquiring sperm motility, and deficiencies in piRNAs are often associated with impaired sperm vitality and morphology. Choi et al. found that mice lacking the pachytene piRNA cluster on chromosome 18 (pi18) exhibited severe sperm abnormalities in mice, including malformed heads, excessive acrosome formation, impaired acrosome exocytosis, defects in the axonemal complex of the mitochondrial sheath, and partial loss of outer dense fibers (ODFs) in the tail (Choi et al., 2021). These abnormalities led to poor sperm motility, reduced activity, and infertility. The absence of pi18 primarily disrupted the structural integrity of the trans-Golgi network, which was associated with enlarged proacrosomal vesicles and upregulation of Golgin subfamily A member 2 (GOLGA2) transcripts and protein (Choi et al., 2021).

Furthermore, Kong et al. discovered that *piR_32362259* may influence the development of sperm damage by regulating the phosphatidylinositol 3-kinase (PI3K) - protein kinase B (AKT) signaling pathway in mice (Kong et al., 2021). Lower expression levels of this piRNA were found to slow the reduction in sperm vitality, influence the cell cycle, and decrease apoptosis rates. In mice, Wu et al. identified that mutations in *pi6* piRNAs led to defective Ca^2+^ influx during the acrosome reaction, impairing the sperm’s ability to penetrate or bind to the zona pellucida, a critical step in fertilization (Wu et al., 2020).

Pachytene piRNAs are critically involved in maintaining sperm motility. The clusters *pi9* and *pi17* serve as major sources of murine pachytene piRNAs, collectively accounting for 13.5% of the total pool. Their loss ablates the production of corresponding pachytene piRNAs and damages RNA cleavage activity (Gainetdinov et al., 2023). Cecchini K et al. generated *pi9* ^*−/−*^ and *pi17* ^*−/−*^ single mutant male mice, which exhibited only mild reductions in sperm motility and remained fertile—indicating genetic redundancy between these loci. In contrast, *pi9* ^*−/−*^ *pi17* ^*−/−*^double mutants displayed severe phenotypes, exhibiting significantly reduced sperm count and motility, loss of zona pellucida penetration ability, and complete infertility (Cecchini et al., 2024). This study underscores the critical role of pachytene piRNAs in regulating sperm function.

**FIGURE 3:**
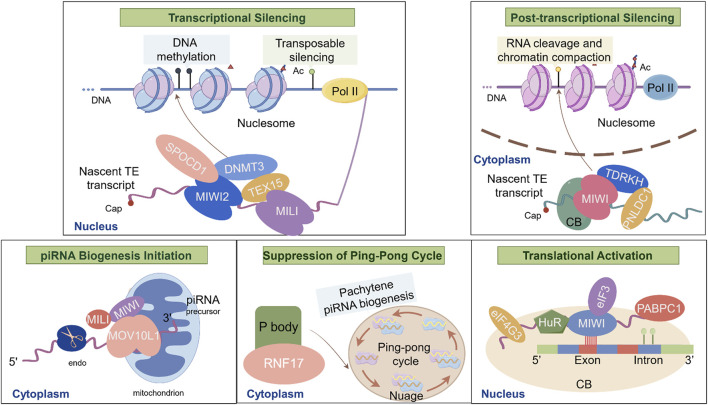
Biological Functions of piRNAs and Major Molecular Mechanisms during spermatogenesis. This schematic illustrates the molecular interactions of piRNAs during spermatogenesis: I. Transcriptional Silencing: TEX15 interacts with MILI to recruit epigenetic silencing complexes (e.g., DNMT3A/3L), targeting LINE1 retrotransposons; SPOCD1 bridges MIWI2 (PIWIL4) with DNMT3L, directing *de novo* DNA methylation at transposon loci to enforce transcriptional silencing; II. Post-transcriptional Silencing: PNLDC1 and TDRKH localize MIWI to chromatoid bodies, facilitating LINE1 RNA cleavage and chromatin compaction in nucleosome; III. piRNA Biogenesis Initiation: MOV10L1 binds to single-stranded piRNA precursors on mitochondria, recruiting MILI and MIWI to initiate primary piRNA processing; IV. Suppression of the Ping-Pong Cycle by ADAD2: ADAD2 activates RNF17 in cytoplasmic P bodies, inhibiting the amplification of secondary piRNAs via the ping-pong cycle in nuage; V. Translational Activation by MIWI/piRNA Complexes: MIWI/piRNA recruits HuR, eIF3, eIF4G3 and PABPC1 to AREs in target mRNAs (e.g., *Plectin*, *Agfg1*), promoting translation essential for acrosome formation in chromtoid bodies.

Additionally, Cornes et al. demonstrated that *C. elegans* spermatocytes lacking PIWI or Argonaute protein HRDE-1 failed to produce pseudopod-like structures observed in wild-type spermatocytes, leading to reduced sperm motility and significantly fewer offspring when crossed with wild-type individuals (Cornes et al., 2022). This highlights the critical role of piRNAs in maintaining sperm motility and ensuring reproductive success. Taken together, specific piRNAs and PIWI/piRNA pathway-associated proteins are involved in different stages of spermatogenesis (Figure 3).

### 3.4 Interplay between piRNAs and hormones during spermatogenesis

To date, direct evidence for the impact of piRNAs on hormonal regulation during spermatogenesis remains limited. While piRNAs have been implicated in modulating hormone-related pathways, their capacity to influence systemic hormone levels or directly regulate endocrine signaling has not been fully elucidated. Current findings suggest a potential role for piRNAs in shaping the hormonal environment of the testis, yet mechanistic insights into this axis are still lacking and warrant further investigation.

Most existing data highlight an association between the piRNA/PIWI pathway and testosterone (TT) signaling. In androgen-deficient rat models, *Miwi* expression is significantly downregulated, whereas follicle-stimulating hormone (FSH) deficiency does not appear to impact *Miwi* levels, implying that *Miwi* is predominantly regulated by TT (Gill-Sharma et al., 2012). Supporting this, Kang et al. observed that in normal rats, both PIWI protein and piRNA expression patterns closely follow TT secretion profiles (Kang et al., 2014). Upon exogenous testosterone treatment, piRNA levels significantly increased across treatment groups, with neonatal-to-juvenile testosterone injection leading to elevated PIWI expression and reduced *Ago3* expression (Kang et al., 2014). These findings suggest that TT may directly modulate the piRNA pathway to influence testicular function.

In addition to androgens, estrogens have also been shown to impact piRNA pathway components. Pan et al. reported that estradiol administration in mice led to a significant downregulation of both *Miwi* and *Mili* at the mRNA and protein levels within the first 3 days of treatment, followed by partial recovery by day 7 (Pan et al., 2012). Notably, this effect was not dose-dependent, and the suppression of *Miwi* expression was more significant than that of *Mili* (Pan et al., 2012). This study provided the first *in vivo* evidence of estrogen-mediated negative regulation of the PIWI pathway in male gonads and suggested a possible antagonistic interaction between estrogen and testosterone in modulating piRNA/PIWI expression.

Corroborating the preclinical findings, clinical data from infertile men further support a link between piRNA dysregulation and hormonal imbalance. Kumar et al., using small RNA sequencing, revealed that men with low serum TT levels (5.9 ± 2.8 nmol/L) displayed distinct piRNA expression profiles, including significant downregulation of piR-hsa-21622 and piR-hsa-28841, compared to other subfertile groups (Kumar et al., 2019). Conversely, in subfertile men with normal TT and FSH levels, hsa-piR-26399 expression was markedly elevated, implying a nuanced relationship between specific piRNA species and hormone status (Kumar et al., 2019).

More recently, Schülke et al. investigated 132 cryptozoospermic patients and 160 men with obstructive azoospermia exhibiting full spermatogenesis (Schulke et al., 2024). Through principal component analysis and hierarchical clustering, they identified that men with Sertoli-cell-only (SCO) phenotype exhibited higher FSH levels, lower TT, reduced testicular volume, and an increased proportion of PIWIL4^+^ spermatogonia (Schulke et al., 2024). PIWIL4 expression was negatively correlated with the presence of elongated spermatids, suggesting that hormonal dysregulation may influence early spermatogonial PIWI expression and contribute to the SCO phenotype (Schulke et al., 2024). These findings not only underscore the clinical relevance of piRNA-hormone interactions but also hint at novel therapeutic avenues for male infertility.

Together, these studies collectively point toward a functional axis between piRNAs and the hormonal environment in the testis, especially involving TT and estrogen signaling. While the mechanistic underpinnings of how hormones modulate piRNA biogenesis and function remain to be fully elucidated, current evidence suggests that piRNAs may act as both effectors and responders of hormonal cues during spermatogenesis. Unraveling this interplay will be essential for advancing our understanding of male fertility and may offer new biomarkers or targets for intervention in male reproductive disorders.

### 3.5 Interplay of piRNAs, miRNAs, and siRNAs

piRNAs, miRNAs, and siRNAs represent three major classes of small non-coding RNAs that collectively regulate gene expression and genomic stability. piRNAs primarily function in the germline by silencing TEs and regulating gene expression. miRNAs are ∼22 nt RNAs that suppress gene expression via mRNA decay and translational repression (Bartel, 2004). siRNAs, characterized as 20–24 nt double-stranded RNAs, function as key mediators of RNA interference (RNAi) (Roberts et al., 2016). While they exhibit distinct biogenesis pathways and primary functions, their regulatory networks share numerous molecular components and exhibit significant cross-talk.

#### 3.5.1 Shared molecular machinery

The Ago family proteins serve as central executors across all three pathways. Ago proteins universally bind small RNAs to form the RNA-induced silencing complex (RISC), mediating gene silencing through translational repression and mRNA decay (Azlan et al., 2016). Certain Ago proteins (e.g., human AGO1/2, *Drosophila* DmAGO2) localize to the nucleus, where they exert additional regulatory functions by promoting DNA methylation and modulating alternative splicing (Tan et al., 2009; Cernilogar et al., 2011; Azlan et al., 2016).

The methyltransferase HEN1 plays a conserved yet pathway-specific role in small RNA stabilization across species. Horwich MD et al. demonstrated that in *Drosophila*, the methyltransferase activity of DmHen1 majorly functions in the piRNA and siRNA pathways (Horwich et al., 2007). DmHen1 critically maintains piRNA stability and function by catalyzing 3′-terminal 2′-O-methylation in germ cells (Horwich et al., 2007). Within the siRNA pathway, DmHen1 similarly methylates the 3′end of single stranded siRNAs following their loading into Ago2-RISC (Horwich et al., 2007). Intriguingly, a subset of Ago2-bound miRNAs (e.g., miR-277) also undergo DmHen1-mediated methylation (Horwich et al., 2007). This functional conservation extends to the *C. elegans* HEN1 ortholog henn-1 (Montgomery et al., 2012). Conversely, in *N. vectensis*, Hen1 primarily methylates miRNAs and piRNAs (Modepalli et al., 2018). Notably, knockdown of PIWI2 in *Nematostella vectensis* not only reduced piRNA levels but unexpectedly caused a significant decrease in miRNA abundance, suggesting piRNA pathway involvement in miRNA homeostasis, potentially via broad transcriptional regulation (Modepalli et al., 2018).

#### 3.5.2 Reciprocal regulation of biogenesis and function

Emerging evidence indicates bidirectional crosstalk between these pathways. In *Drosophila*, Iki T et al. demonstrated that siRNAs can directly initiate piRNA biogenesis: 3′fragments generated by siRNA-directed cleavage of ATPsynbeta mRNA are loaded onto Aub and processed into CDS-piRNAs, evidenced by 5′-end complementarity between the siRNAs and resultant piRNAs (Iki et al., 2023). Furthermore, miRNAs (e.g., miR-316) bound to Ago2 can specify piRNA precursor regions by recognizing antisense sequences within target transcripts (e.g., muc14A), positioning Ago2 to initiate CDS-piRNA biogenesis (Iki et al., 2023).

miRNAs and piRNAs engage in reciprocal regulatory loops critical for developmental processes. Du WW et al. revealed that during murine embryonic development, miR-17-5p disrupts TE silencing by inhibiting the piRNA ping-pong amplification cycle and downregulating *Mili* and *Dnmt3a*. Conversely, injection of piR-11 into zygotes competitively attenuated miR-17-5p-mediated suppression of *Mili* and *Dnmt3a*, demonstrating mutual regulation essential for normal embryogenesis and TE control (Du et al., 2016).

piRNA-siRNA cooperation is evolutionarily conserved for robust gene silencing. High-throughput sequencing in four nematodes (*C. elegans*, *Caenorhabditis briggsae*, *Caenorhabditis remanei, Caenorhabditis brenneri*) revealed a conserved mechanism where piRNAs guide AGO proteins to cleave target mRNAs, triggering RdRP-dependent amplification of secondary siRNAs (Shi et al., 2013). In *C. elegans*, Manage KI et al. elucidated that piRNAs bound to the PIWI homolog PRG-1 recognize target mRNAs within P granules (Manage et al., 2020). This initial recognition, facilitated by the Tudor domain protein SIMR-1, nucleates secondary siRNA biogenesis, establishing a synergistic “piRNA-priming, siRNA-amplification” cascade essential for silencing target mRNAs, maintaining genomic integrity, and ensuring proper germ cell development (Manage et al., 2020).

piRNAs, miRNAs, and siRNAs form an interconnected regulatory network through shared biogenesis factors, reciprocal modulation, and overlapping effector mechanisms. These intricate interactions are crucial for fine-tuning gene expression, particularly during germline development and early embryogenesis. Understanding these relationships is fundamental to elucidating how small RNA pathways coordinate to maintain genomic integrity and regulate complex developmental programs.

## 4 piRNAs and clinical conditions

The piRNA pathway plays a crucial role in male reproductive health, and its dysfunction is linked to infertility. Understanding the dynamic functions of piRNAs at different stages of spermatogenesis is essential for developing novel therapeutic strategies for male infertility (He et al., 2022; Wang et al., 2021) (Table 3).

**TABLE 3:** Functions of piRNAs at Different Stages of Spermatogenesis.

piRNA	Species	Functions in testis	Ref.
Mitosis of spermatogonia			
piR_003399	*Mus musculus*	exert specific cytotoxic effects on spermatogonia lead to cell cycle arrest, mature sperm and motility reduction, and abnormal sperm morphology caused by MC-LR	Zhang et al. (2018)
piR-020492	*Mus musculus*	be upregulated under testis heat stress inhibit the proliferation and promote apoptosis of germ cells by affecting AMPK/insulin pathway	Chen et al. (2023)
CDS-piRNAs	*Drosophila melanogaster*	form a silencing complex AUB-RISC with Aub regulate endogenous gene expression influence post-transcriptional modification of histones and their acetylation levels	Iki et al. (2023)
Meiosis of the spermatocytes			
pi6 piRNAs	*Mus musculus*	trim pachytene piRNA precursor transcripts to initiate piRNA production promote biogenesis of piRNAs from other sites repeatedly be related to sperm capacitation	Wu et al. (2020)
pi10-qC2-545.1 piRNAs	*Mus musculus*	promote the biogenesis of pi6 piRNA	Wu et al. (2020)
Maturation of the round spermatids			
piR-12	*Mus musculus*	promote affinity between HuR and MIWI/eIF3f complex with Spesp1 reporter to activate translation	Iki et al. (2023)
CDS-piRNAs	*Drosophila melanogaster*	form a silencing complex AUB-RISC with Aub influence post-transcriptional modification of histones and their acetylation levels	Iki et al. (2023)
piR_1029	*Mus musculus*	promote affinity between HuR and MIWI/eIF3f complex through piRNA:mRNA and translational activation complete the assembly of the MIWI/eIF 3f/HuR supercomplex in round sperm cells upregulate target reporters Plectin, Agfg1, Tbpl1, Cnot4, and Atg12	Dai et al. (2019)
piR_010111 piR_3146 piR_2617, piR_3344	*Mus musculus*	inhibit some genes promote affinity between HuR and MIWI/eIF3f complex through piRNA:mRNA upregulate target reporters Plectin, Agfg1, Tbpl1, Cnot4, and Atg12	Dai et al. (2019)
piR_32362259	*Mus musculus*	regulate PI3K-AKT signaling pathway to affect the occurrence and development of sperm injury	Kong et al. (2021)
pi18	*Mus musculus*	maintain sperm morphology	Choi et al. (2021)
piR_121380	*Sus scrofa domesticus*	downregulate PTPN7 mainly concentrated in the sperm head to increase the phosphorylation of ERK2 and the motility and activation rate of sperm during cryopreservation	Wang et al. (2021)
piR-1207, piR-2107, piR-31068 piR-31925, piR-43771, piR-43773	*Homo sapiens*	be potential diagnostic targets of asthenospermia	Hong et al. (2016)
piR-hsa-32694, piR-hsa-26591 piR-hsa-18725, piR-hsa-18586	*Homo sapiens*	be potential diagnostic targets of asthenospermia	He et al. (2022)

### 4.1 piRNAs and their clinical potentials as biomarkers

Due to their germ cell origin, decrease piRNA levels in sperm and seminal plasma may serve as biomarkers of reduced germ cell populations, particularly in azoospermic and oligozoospermic men (Larriba et al., 2023). Hong et al. reported significantly lower expression of piR-1207 and piR-2107 in the seminal plasma and sperm of oligozoospermic men, along with a general reduction in piRNA diversity and abundance (Hong et al., 2016). Moreover, Receiver Operating Characteristic (ROC) curve analysis identified five piRNAs (piR-31068, piR-31925, piR-43771, piR-30198 and piR-43773) as potential biomarkers for non-obstructive azoospermia. Notably, piR-30198 exhibits exclusive downregulation in azoospermia and may serve as a specific diagnostic biomarker distinguishing azoospermia from asthenozoospermia (Hong et al., 2016). Asthenozoospermia is a condition characterized by reduced sperm motility (Shahrokhi et al., 2020). Barceló M et al. compared exosomal microRNAs in seminal plasma exosomes between healthy individuals and patients with obstructive azoospermia to identify a significant downregulation of piR-58527 in the seminal plasma exosomes of obstructive azoospermia patients. This specific piRNA demonstrated precise diagnostic accuracy with high sensitivity and specificity (Barcelo et al., 2018). These findings demonstrate the potential of seminal plasma piRNAs as novel molecular biomarkers for the diagnosis of both non-obstructive azoospermia and obstructive azoospermia.

piRNAs demonstrate diagnostic potential in patients with asthenozoospermia. Hong Y et al. observed no significant alterations in the size or concentration of seminal plasma exosomes between asthenozoospermia patients and normozoospermic controls. However, they identified marked downregulation of piR-1207 and piR-2107 within these exosomes, concomitant with significantly reduced MitoPLD protein expression in spermatozoa in asthenozoospermia patients (Hong et al., 2021). These findings suggest that MitoPLD dysfunction may contribute to asthenozoospermia pathogenesis through impaired piRNA biogenesis. Furthermore, piR-1207 and piR-2107 exhibit promise as diagnostic biomarkers for asthenozoospermia, demonstrating high sensitivity and specificity, though validation in larger cohorts is warranted given the preliminary sample size (Hong et al., 2021). In a further study, He L et al. employed small RNA sequencing and reverse transcription polymerase chain reaction (RT-qPCR) validation to discover significant upregulation of piR-hsa-32694, piR-hsa-26591, piR-hsa-18725, and piR-hsa-18586 in asthenozoospermia patients. ROC analysis revealed exceptional diagnostic performance: piR-hsa-26591 achieved an Area Under the Curve (AUC) of 0.913, while the four-piRNA joint diagnosis model reached an AUC of 0.935 (He et al., 2022).

A clinically significant subset of non-obstructive azoospermia patients exhibits focal spermatogenic activity within the testicular parenchyma. For these individuals, microdissection testicular sperm extraction (micro-TESE) represents a critical surgical intervention, enabling the retrieval of viable sperm from localized regions of preserved spermatogenesis. Contemporary clinical evidence indicates procedure success rates approximating 40% in this patient cohort (Vahidi et al., 2021; Achermann et al., 2021). piRNAs may act as biomarkers in assessment for male infertility treatments. Cao C et al. performed comparative profiling of testicular piRNA expression between successful and unsuccessful micro-TESE groups in non-obstructive azoospermia patients (Cao et al., 2018). Their analysis revealed significant downregulation of 20 piRNAs in the micro-TESE failure group. Notably, hsa-piR-6254 and hsa-piR-17765 exhibited comparable expression levels in seminal plasma and testicular tissue, demonstrating their potential as non-invasive biomarkers for predicting micro-TESE outcomes (Cao et al., 2018). Chen H et al. identified significant downregulation of 8 piRNAs (piR-31704, piR-31843, piR-36659, piR-45048, piR-46102, piR-55522, piR-60351, and piR-61927) in extracellular vesicles (EVs) derived from seminal plasma of non-obstructive azoospermia patients (Chen H. et al., 2021). piR-61927 within seminal plasma EVs also demonstrated efficacy as a biomarker for predicting micro-TESE outcomes (Chen H. et al., 2021). Its non-invasive nature, high diagnostic accuracy (AUC = 0.83), and single-marker predictive capability establish this piRNA as a novel standardized tool for preoperative assessment in non-obstructive azoospermia, potentially reducing unnecessary surgical interventions. However, as a single-center pilot-scale investigation, these findings require validation through multicenter studies with expanded cohorts. Furthermore, developing composite biomarker panels (e.g., integrating piRNAs with hormonal profiles) may enhance predictive performance beyond current capabilities.

Spermatozoal piRNA levels may correlate with intracytoplasmic sperm injection (ICSI) success rates. Cui L et al. compared piRNA expression in spermatozoa between normozoospermic controls and patients with idiopathic male infertility undergoing first ICSI cycles. The infertile cohort exhibited significantly reduced levels of piR-31704 and piR-39888. Conversely, subgroups achieving high 2 pronuclei (2 PN) formation rates demonstrated elevated expression of piR-31704, piR-39888, and piR-40349. However, no significant associations emerged between piRNA levels and conventional semen parameters (motility, morphology), early embryo cleavage rates, top-quality embryo formation, or clinical pregnancy outcomes (Cui et al., 2018). These findings indicate that specific sperm piRNAs may associate with sperm concentration and ICSI fertilization competence, suggesting potential as biomarkers for fertilization potential assessment. Nevertheless, ROC curve analysis revealed suboptimal diagnostic performance for these piRNAs in detecting male infertility (AUC <0.75 for all three), indicating limited immediate clinical utility as standalone diagnostic markers (Cui et al., 2018).

### 4.2 Genetic and epigenetic alterations in key piRNA biogenesis genes

Mutations in genes involved in the piRNA pathway are frequently linked to male reproductive disorders. Genetic defects in the piRNA biogenesis pathway led to transposon de-repression, which contributes to spermatogenic failure and male infertility. Stallmeyer et al. conducted whole-exome sequencing on 412 idiopathic infertile men identified and identified biallelic pathogenic/likely pathogenic (P/LP) variants in 14 piRNA pathway genes (e.g., *PNLDC1, TDRKH, FKBP6*); Functional validation in *Drosophila* models confirmed that these mutations disrupted transposon silencing and triggered germ cell apoptosis, ultimately leading to infertility (Stallmeyer et al., 2024).

Among these genes, *PNLDC1* plays a pivotal role in piRNA maturation. Nagirnaja et al. identified mutations in *PNLDC1* in four unrelated men with non-obstructive azoospermia, demonstrating reduced expression of PIWIL1, PIWIL4, and other key piRNA pathway proteins in testicular tissue (Nagirnaja et al., 2021). Similarly, Li et al. discovered a homozygous *PNLDC1* variant in a Han Chinese patient with severe oligoasthenoteratozoospermia through exome sequencing of 456 infertile patients (Li et al., 2022). Oligoasthenoteratozoospermia is one of the common diseases leading to male infertility, characterized by low sperm concentration, decreased motility and low percent of morphologically normal sperm (Colpi et al., 2018). In the same study, two compound heterozygous variants and a biallelic missense variant in *MOV10L1* were identified in patients with non-obstructive azoospermia or severe oligozoospermia (Li et al., 2022). Oligozoospermia is characterized by a sperm concentration below the normattive threshold of 15 million sperm per milliliter of ejaculate observed in healthy males (Choy and Amory, 2020). Sperm concentrations ≤1 million/mL is considered as severe oligozoospermia (Bak et al., 2010). These findings suggest that mutations in key piRNA biogenesis proteins may contribute to dyszoospermia.

**FIGURE 4:**
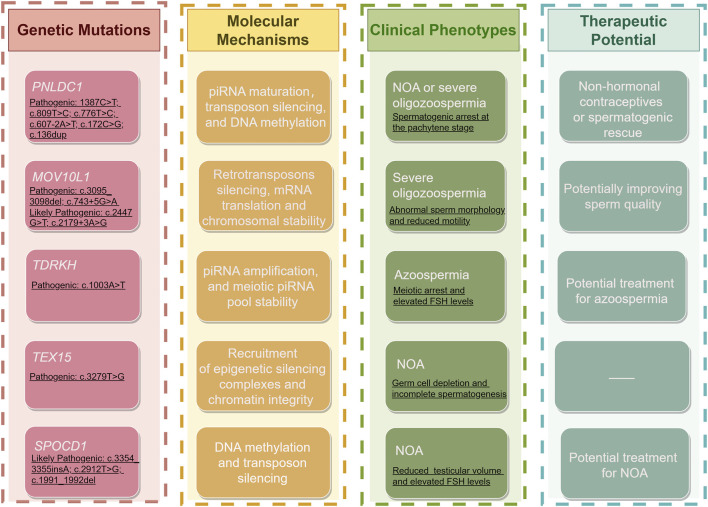
Genetic Defects in piRNA Pathway Genes and Their Implications in Male Infertility. This schematic summarizes the roles of key piRNA biogenesis genes (*PNLDC1, MOV10L1, TDRKH, TEX15*, and *SPOCD1*) in male infertility, integrating genetic, molecular, clinical, and therapeutic perspectives. I. Genetic Mutations: Pathogenic and likely pathogenic variants in these five key piRNA biogenesis genes are linked to male infertility. II. Molecular Mechanisms: (1) *PNLDC1*: Loss of piRNA trimming activity impairs piRNA maturation, leading to LINE1/IAP transposon reactivation and aberrant DNA methylation. (2) *MOV10L1*: Defective piRNA processing results in mRNA translation errors and chromosomal instability due to unsilenced retrotransposons. (3) *TDRKH*: Impaired piRNA amplification destabilizes meiotic piRNA pools. (4) *TEX15:* Failed recruitment of epigenetic silencing complexes (e.g., MILI) permits transposon mobilization, disrupting chromatin integrity. (5) *SPOCD1*: Compromised piRNA-guided DNA methylation at transposon loci reactivates retroelements. III. Clinical Phenotypes: Shared outcomes include non-obstructive azoospermia (NOA), oligo/azoospermia, spermatogenic arrest, abnormal sperm morphology, reduced motility and elevated FSH levels. IV. Therapeutic Potential: Genetic defects in *PNLDC1, MOV10L1, TDRKH, TEX15*, and *SPOCD1* present actionable targets for male infertility treatment. (1) *PNLDC1:* Pharmacological modulation may restore piRNA maturation, offering potential for non-hormonal contraceptives or spermatogenic rescue. (2) *MOV10L1*: Correcting its function could re-establish piRNA biogenesis, improving sperm quality (3) *TDRKH:* Gene-editing strategies (e.g., CRISPR). to restore transposon silencing may address azoospermia (4) *TEX15*: Epigenetic regulators (e.g., DNMT inhibitors). could compensate for defective transposon repression. (5) *SPOCD1*: DNA methyltransferase enhancers might mitigate transposon activity in non-obstructive azoospermia.

Disruptions in the PIWI/piRNA pathway contribute to male infertility through several mechanisms (Figure 4): (i) Reactivation of transposons, causing chromosomal instability and cell death; (ii) Spermatogenic arrest from disrupted gene expression and DNA methylation; (iii) Defective sperm morphology and reduced motility; (iv) Lower sperm count due to deficiencies in key proteins like MOV10L1; and (v) Altered chromatin structure, affecting sperm DNA integrity.

### 4.3 Impact of other piRNA-related genes

Beyond core piRNA biogenesis genes, mutations in piRNA-associated genes have been linked to male infertilitys (Table 2). FKBP6, which participates in secondary piRNA biogenesis, has been found to harbor biallelic loss-of-function (LoF) variants (e.g., p.Arg278Cys, LP) in multiple infertile men, leading to severe oligozoospermia or azoospermia with abnormal sperm morphology (Stallmeyer et al., 2024). Similarly, *SPOCD*—a key regulator of transposon DNA methylation—has been associated with non-obstructive azoospermia and elevated FSH levels when harboring pathogenic variants such as p.Gln1119fs (Zoch et al., 2024).

Other notable genes include *HENMT1*, a regulator of piRNA methylation, and the *TDRD* family, which is essential for arginine methylation and piRNA function. It is noteworthy that the role of HENMT1 in piRNA methylation has thus far been exclusively documented in humans and mice, with no analogous function observed in *Drosophila*. Homozygous LoF variants in *TDRD*9 (p.Gly1276Cys, variant of uncertain significance (VUS)) and *TDRKH* (p.Lys335Ter, P) have been linked to human azoospermia (Kherraf et al., 2022). Moreover, *TEX15*—which collaborates with *MILI* to recruit epigenetic silencing machinery to transposon loci—is crucial for transposon suppression in mice. Okutman et al. identified a homozygous premature stop codon in *TEX15* in two infertile brothers from a consanguineous Turkish family (Okutman et al., 2015). Likewise, Gou et al. screened 413 men with oligozoospermia and azoospermia and identified D-box heterozygous mutations in *HIWI* in three individuals. Pedigree analysis suggested that these mutations were either maternally inherited or arose *de novo* (Gou et al., 2017).

### 4.4 Treatment protocols and therapeutic potential

To date, treatment protocols for mutations in piRNA-related genes have been largely confined to TESE or micro-TESE, followed by ICSI, with generally poor outcomes. Four *PNLDC1* variant carriers with non-obstructive azoospermia underwent underwent TESE/micro-TESE, with no successful pregnancies achieved (Nagirnaja et al., 2021). Stallmeyer et al. performed TESE on 23 of 39 infertile men carrying biallelic variants in 14 distinct piRNA pathway genes. Spermatozoa were retrieved solely in one patient with a *TDRD9* variant, yielding extremely few spermatozoa exhibiting abnormal morphology and severely impaired motility. Subsequent ICSI in this *TDRD9* variant carrier failed to achieve pregnancy, attributable to compromised sperm morphology, impaired fertilizing capacity, and/or embryonic developmental arrest (Stallmeyer et al., 2024). Wyrwoll et al. reported six individuals with *FKBP6* variants exhibiting oligozoospermia or azoospermia. Following fine needle aspiration (FNA), retrieved spermatozoa displayed severe morphological abnormalities (e.g., multiflagellate spermatozoa, head defects, cytoplasmic fragmentation), rendering them unsuitable for assisted reproductive technology (ART) procedures (Wyrwoll et al., 2022). Three infertile men carrying homozygous pathogenic *SPOCD1* variants presented with non-obstructive azoospermia. TESE procedures also failed to retrieve usable spermatozoa in all cases (Zoch et al., 2024). piRNA biogenesis protein EXD1 is a protein containing an exonuclease domain that, along with MIWI2 and TDRD12, is involved in secondary piRNA biogenensis and TE silencing in mammalian germ cells (Yang et al., 2016). Hu K et al. reported the first documented LoF variant in the *EXD1* gene, identified in a patient presenting with oligoasthenoteratozoospermia characterized by acrosomal hypoplasia. The patient subsequently achieved a healthy live birth following ICSI treatment (Hu et al., 2025).

Notably, Akbari et al. reported a potential therapeutic approach for asthenozoospermia associated with one *Adenylate cyclase type 10 (ADCY10)* mutation. Supplementation of exogenous cyclic adenosine monophosphate (cAMP) analogue (dibutyryl cAMP sodium salt), dissolved in Human Tubal Fluid (HTF) to prepare a 20 mM stock solution, was employed to elevate cAMP levels, thereby activating the downstream PKA pathway and restoring sperm motility (Akbari et al., 2019). The ultimate pregnancy outcome for this specific patient was not reported. For similar cases of *ADCY10* mutation presenting with normal sperm count but impaired motility, a recommended strategy involves *in vitro* treatment of spermatozoa with cAMP analogue (recommended dose: 0.05 mg/mL, 30-min incubation) followed by ICSI. For patients exhibiting spermatogenic arrest at the round spermatid stage (e.g., some carriers of FKBP6 or PNLDC1 mutations), round spermatid injection (ROSI) represents a theoretical option. However, ROSI currently demonstrates very low clinical success rates and lacks robust evidence supporting its efficacy (Hanson et al., 2021).

ART targeting oocyte activation may represent a promising therapeutic avenue for male infertility caused by piRNA pathway deficiencies. Guo R et al. documented a seminal case involving a *TDRD5* mutation in two homozygous variant carriers from China presenting with severe oligoasthenoteratozoospermia (Guo et al., 2025). Sperm from both patients exhibited multiple morphological abnormalities, including multi-headed/multi-flagellated sperm and acrosomal malformations. Testicular analysis revealed significantly reduced expression of key IMC/CB components PIWIL1 and regulator of nonsense transcripts 1 (UPF1), along with a marked decrease in pachytene piRNA abundance. Following failed initial ICSI cycles, both patients underwent a modified protocol combining ICSI with artificial oocyte activation (AOA) using 10 µM calcium ionophore A23187 for 5 min. This intervention elevated fertilization rates to ≥50%, culminating in a live birth in one case (Guo et al., 2025). These outcomes suggest that AOA may offer therapeutic potential for male infertility linked to piRNA-related acrosomal defects.

Environmental stressors, such as oxidative stress and endocrine disruptors, further exacerbate infertility by impairing piRNA-mediated transposon silencing in gonad cells and germ cells. This underscores the urgent need for research into infertility treatments targeting this pathway (Munzel and Daiber, 2018). piRNAs may function as natural tumor suppressors in humans. Yuan J et al. discovered that piR-26441 directly binds to and upregulates *YTH domain-containing protein 1 (Ythdc1)* expression, which increases m6A modification to destabilize *elongation factor Ts (Tsfm)* mRNA (Yuan et al., 2025). This leads to reduced *Tsfm* expression, thereby suppressing mitochondrial oxidative phosphorylation (OXPHOS) and decreasing Complex I activity in human mitochondrion. Consequently, mitochondrial dysfunction and reactive oxygen species (ROS) accumulation occur, resulting in DNA damage and apoptosis. This study links piRNAs with oxidative stress, indicating that piRNAs may serve as a promising diagnostic biomarker for OXPHOS-associated ovarian cancer (Yuan et al., 2025).

Notably, piRNA-mediated interference (piRNAi) offers a precise and scalable gene-silencing strategy. Priyadarshini et al. demonstrated that in *C. elegans*, piRNAi enables targeted gene regulation via auxin-mediated degradation of PRG-1, leading to transgenerational epigenetic silencing (Priyadarshini et al., 2022). Similarly, Fabry et al. engineered a rapidly degrading PIWI protein after embryo formation in *Drosophila*, providing insights into the role of maternally deposited PIWI proteins in transposon regulation (Fabry et al., 2021). This strategy could be adapted to study the effects of sperm-derived PIWI proteins on embryogenesis.

As previously established, piRNAs demonstrate diagnostic potential for male infertility and micro-TESE outcome prediction. However, current biomarker researches on piRNAs remain predominantly focused on oncological applications. Multiple piRNAs, including piR-9491, piR-018569, piR-5937, piR-28876, piR-020619, and piR-020450, have been validated as high-performance diagnostic biomarkers across gastrointestinal and renal malignancies. These molecules consistently demonstrate superior sensitivity and robust AUC values, frequently outperforming established biomarkers in both early detection accuracy and diagnostic specificity (Wang Z. et al., 2020; Vychytilova-Faltejskova et al., 2018; Sohn et al., 2023). In stark contrast, the clinical translation of piRNA as biomarkers for male infertility diagnostics and treatments demands expanded mechanistic investigation and multi-center validation studies.

Overall, therapeutic interventions targeting the piRNA pathway hold significant promise for treating male infertility. Strategies such as modulating RNF8-MIWI interactions or targeting PNLDC1 could lead to novel fertility treatments. Additionally, the piRNA pathway may offer innovative approaches to non-hormonal contraception, broadening reproductive health options. As research advances, a deeper understanding of piRNA dynamics in testicular tissues and spermatozoa could pave the way for improved fertility treatments and reproductive health strategies.

## 5 Discussion, limitations and further perspectives

The piRNA pathway emerges as a central regulatory axis in spermatogenesis, with its dysfunction intricately linked to male infertility. This review consolidates evidence from animal models and human genetic studies, demonstrating that inherited defects in piRNA biogenesis genes (e.g., *PNLDC1*, *MOV10L1*, *TDRD9*) disrupt transposon silencing, induce genomic instability, and impair germ cell differentiation. Notably, mutations in these genes are strongly associated with clinical phenotypes such as non-obstructive azoospermia and oligoasthenoteratozoospermia, underscoring the essential role of piRNA pathway in male fertility.

A key advancement highlighted here is the identification of piRNAs as diagnostic biomarkers in seminal plasma (e.g., piR-hsa-26591 for asthenozoospermia) and spermatozoa, offering non-invasive tools for male infertility evaluation. Furthermore, the stage-specific dynamics of PIWI proteins (such as MIWI’s role in chromatin compaction during spermiogenesis and MIWI2’s nuclear localization for TE methylation) reveal spatiotemporal precision in piRNA-mediated regulation. These findings bridge gaps between animal studies and human pathology, particularly in explaining how piRNA defects lead to meiotic arrest or sperm structural abnormalities.

However, limitations persist. While knockout models (e.g., *Piwil1* ^*−/−*^ mice) robustly recapitulate human infertility phenotypes, translational challenges remain. For instance, the functional validation of rare human variants (e.g., HIWI mutations) is often constrained by limited patient-derived germline samples. Additionally, the interplay between piRNAs and environmental stressors (e.g., oxidative stress, endocrine disruptors) warrants deeper exploration, as these factors may exacerbate piRNA pathway dysregulation in clinical settings.

Current evidence indicates that therapeutic options for individuals carrying pathogenic variants in piRNA-related genes remain limited, often yielding suboptimal outcomes. A critical gap exists in the widespread lack of precisely targeted therapies directed against the piRNA pathway. While exogenous administration of cAMP analogues represents a potential targeted strategy for infertility caused by ADCY10 mutations, this approach has not advanced to clinical trials. Strategies such as modulating PIWI protein interactions (e.g., targeting the RNF8-MIWI complex), targeting key piRNA pathway components (e.g., PNLDC1, MOV10L1), or utilizing piRNAi hold promise for novel fertility treatments. However, their development is currently only confined to preclinical animal models. Although the piRNA pathway exhibits a high degree of evolutionary conservation, rigorous experimental validation is essential to establish the efficacy and feasibility of these therapeutic concepts. Furthermore, while emerging clinical data suggest piRNA expression levels may serve as a biomarker for the extent of environmentally induced male fertility impairment, comprehensive clinical data collection and further investigation are imperative. Additionally, the piRNA pathway may offer innovative approaches to non-hormonal contraception, broadening reproductive health options. As research advances, a deeper understanding of piRNA dynamics in testicular tissues and spermatozoa could pave the way for improved fertility treatments and reproductive health strategies.

The therapeutic potential of piRNA pathway has taken initial steps towards clinical translation from foundational research. However, significant practical hurdles remain. First, interspecies divergences between animal models and humans (e.g., species-specific TE silencing mechanisms) pose substantial challenges for extrapolating preclinical findings. Most interventional strategies remain confined to *in vitro* or preclinical animal models. Future progress will necessitate large-scale clinical validation and the refinement of targeted delivery systems (e.g., spermatogonia-specific vectors) to advance piRNA-based therapies. Second, delivery obstacles impede the development of piRNA therapeutics, including *in vivo* instability, off-target effects, and inadequate tissue-specific targeting. A primary physiological barrier is the blood-testis barrier (BTB), which rigorously regulates molecular transit into the seminiferous tubules. Composed of tight junctions, gap junctions, and basement membranes between adjacent Sertoli cells, the BTB maintains the spermatogenic microenvironment and protects germ cells from immune attack (Du et al., 2023). This highly selective barrier excludes most exogenous agents, including nucleic acid-based therapeutics and their delivery vehicles, from reaching target cells (e.g., spermatogonia, spermatocytes). Nanostructured lipid carriers (NLCs) represent a promising delivery vehicle candidate due to their demonstrated capacity to BTB, yet their therapeutic applicability requires further investigation (Tanyapanyachon et al., 2023). Direct supplementation of piRNAs or piRNA pathway components may disrupt epigenetic regulation in both germ and non-germ cells (e.g., Leydig cells, Sertoli cells), potentially leading to secondary spermatogenic defects or even testicular germ cell tumors. Moreover, piRNA functionality depends critically on intact secondary structure (Balaratnam et al., 2019). Certain delivery vehicles (e.g., cationic polymers) may compromise piRNA integrity, preventing formation of functional pi-RISCs or accelerating degradation, thereby abolishing *in vivo* activity. Third, safety concerns regarding germline gene editing technologies warrant careful consideration, particularly given ongoing ethical debates. Finally, large-scale, validated clinical cohorts are urgently needed to establish piRNA-based biomarkers with robust sensitivity and specificity for male infertility.

Future directions should prioritize:

(i) Mechanistic studies: Elucidating post-transcriptional roles of pachytene piRNAs in mRNA translation and their crosstalk with ubiquitin-proteasome systems.
(ii) Therapeutic innovation: Developing small-molecule inhibitors (e.g., targeting RNF8-MIWI interactions) or gene-editing strategies to restore piRNA function in defective germ cells.
(iii) Clinical translation: Validating piRNA biomarkers in large cohorts and exploring non-hormonal contraceptives via PNLDC1 modulation.
(iv) Environmental interactions: Investigating how pollutants or metabolic stressors impair piRNA networks, informing preventive strategies.
