# Correction: Cracking the code: how piRNA pathway shapes spermatogenesis and combats male infertility

**DOI:** 10.3389/fcell.2025.1710695

**Published:** 2025-10-06

**Authors:** Zhidan Hong, Sihan Huang, Li Li, Ying Gao, Binyu Ma, Qigang Fan, Yuanzhen Zhang, Mei Wang

**Affiliations:** ^1^ Center for Reproductive Medicine, Zhongnan Hospital of Wuhan University, Wuhan, Hubei, China; ^2^ Clinical Medicine Research Center of Prenatal Diagnosis and Birth Health in Hubei Province, Wuhan, Hubei, China; ^3^ Wuhan Clinical Research Center for Reproductive Science and Birth Health, Wuhan, Hubei, China; ^4^ Second Clinical Hospital of Wuhan University, Wuhan, Hubei, China

**Keywords:** PIWI proteins, spermatogenesis, male infertility, transposable elements, epigenetic regulation, piRNA biomarkers

Author **Li Li** was erroneously omitted as equal contributing author.

The original article has been updated.

